# Supplementary material for: Spatial transcriptomics deconvolution at single-cell resolution using Redeconve
Source: Nat Commun. 2023 Dec 1;14:7930. doi: 10.1038/s41467-023-43600-9 (PMC10692090; doi:10.1038/s41467-023-43600-9)
Supplement: Supplementary file 1 — Supplementary Information [file 41467_2023_43600_MOESM1_ESM.pdf]

Supplementary Materials for

Spatial transcriptomics deconvolution at single-cell resolution using Redeconve

Zixiang Zhou, Yunshan Zhong, Zemin Zhang, Xianwen Ren*

*Corresponding author. E-mail: renxwise@cpl.ac.cn

This PDF file includes:
Supplementary Figures S1 to S35
Supplementary Tables S1 to S4

Contents

Supplementary Figures 2
Supplementary Fig 1. 2
Supplementary Fig 2. 3
Supplementary Fig 3. 5
Supplementary Fig 4. 6
Supplementary Fig 5. 7
Supplementary Fig 6. 8
Supplementary Fig 7. 9
Supplementary Fig 8. 10
Supplementary Fig 9. 11
Supplementary Fig 10. 12
Supplementary Fig 11. 13
Supplementary Fig 12. 14
Supplementary Fig 13. 15
Supplementary Fig 14. 16
Supplementary Fig 15. 18
Supplementary Fig 16. 19
Supplementary Fig 17. 20
Supplementary Fig 18. 21
Supplementary Fig 19. 22
Supplementary Fig 20. 23
Supplementary Fig 21. 24
Supplementary Fig 22. 25
Supplementary Fig 23. 27
Supplementary Fig 24. 28
Supplementary Fig 25. 29
Supplementary Fig 26. 30
Supplementary Fig 27. 31
Supplementary Fig 28. 33
Supplementary Fig 29. 34
Supplementary Fig 30. 35
Supplementary Fig 31. 36
Supplementary Fig 32. 37
Supplementary Fig 33. 38
Supplementary Fig 34. 39
Supplementary Fig 35. 42
Supplementary Tables 43
Supplementary Table S1. 43
Supplementary Table S2. 44
Supplementary Table S3. 45
Supplementary Table S4. 46

Supplementary Figures

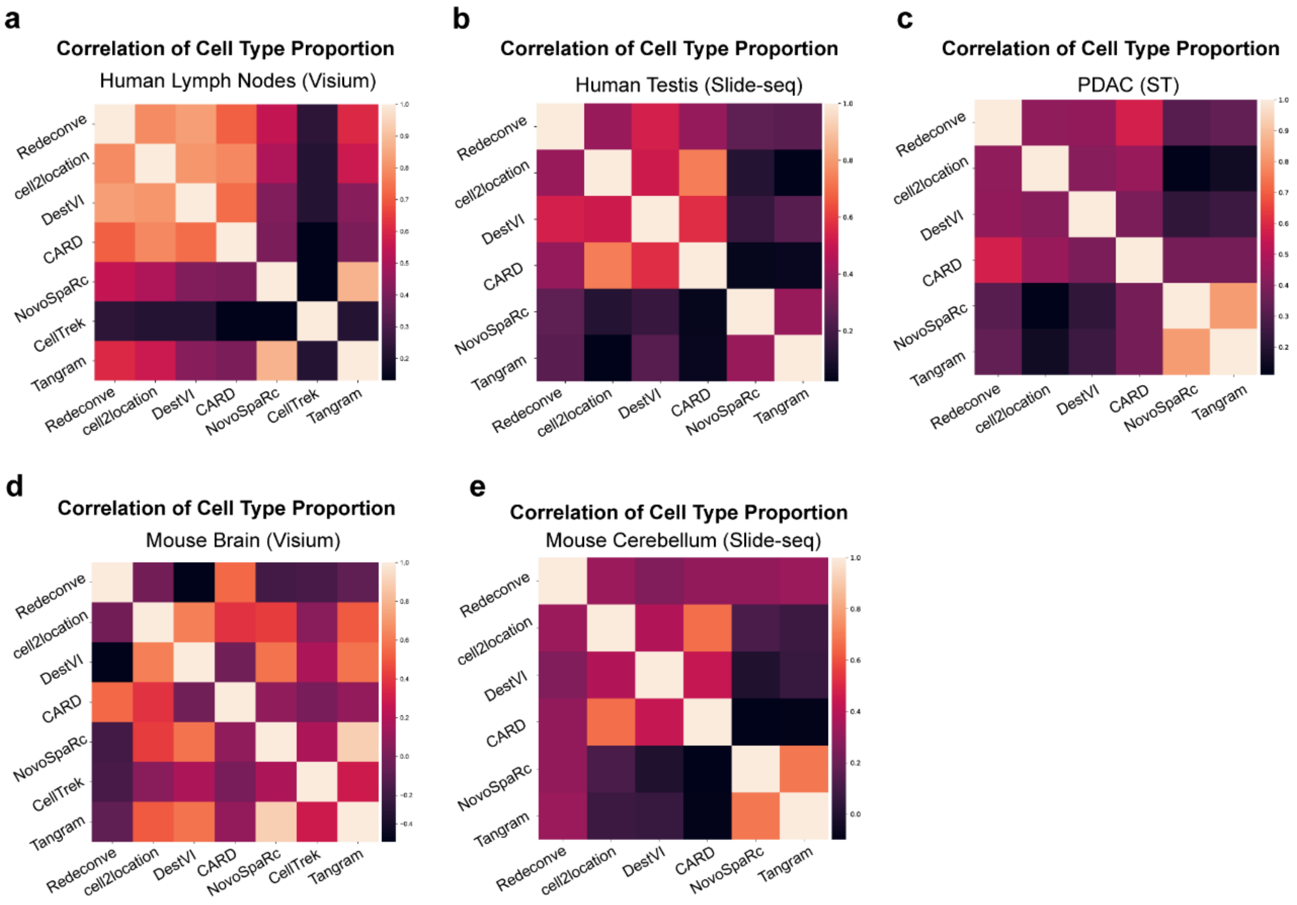

Supplementary Fig 1. Heatmap illustrated median spot-level Spearman's correlations of cell type proportions among different algorithms on five datasets. a, Human Lymph Nodes. b, Human Testis. c, PDAC (pancreatic ductal adenocarcinoma). d, Mouse Brain. e, Mouse Cerebellum. Source data are provided as a Source Data file.

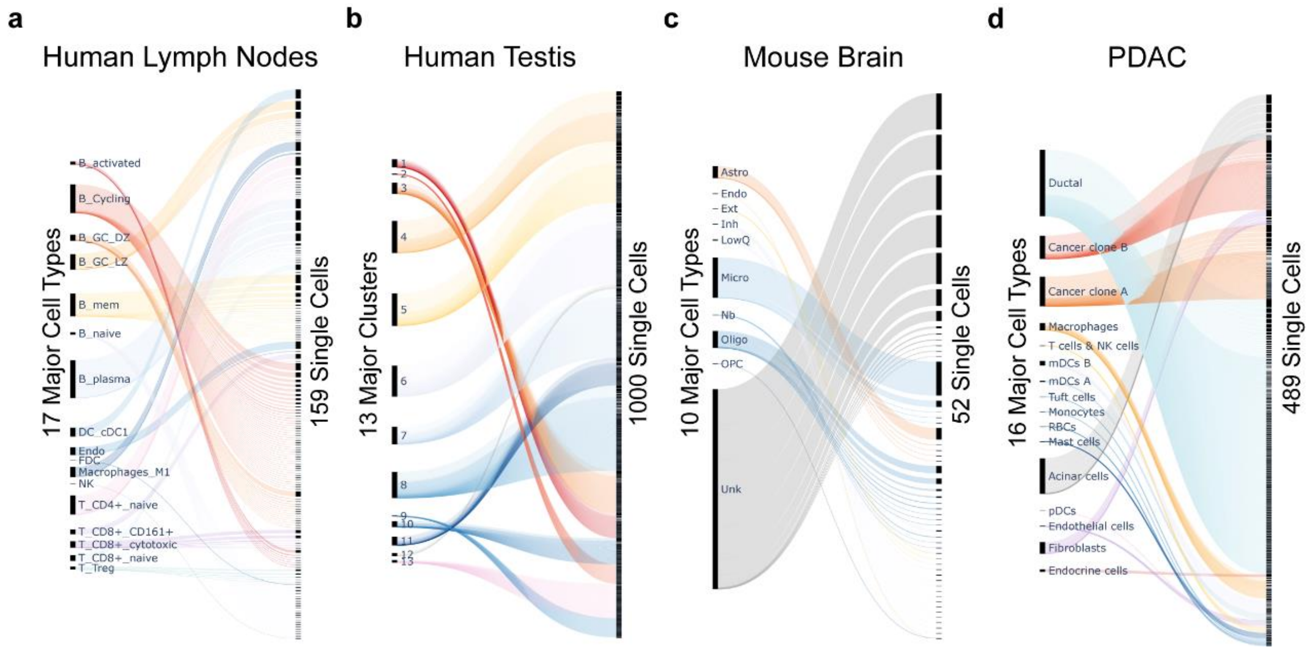

Supplementary Fig 2. Sankey diagram demonstrated the cell-type and single-cell resolutions of Redeconve results on four datasets. a, Human Lymph Nodes. b, Human Testis. c, Mouse Brain. d, PDAC (pancreatic ductal adenocarcinoma). Source data are provided as a Source Data file.

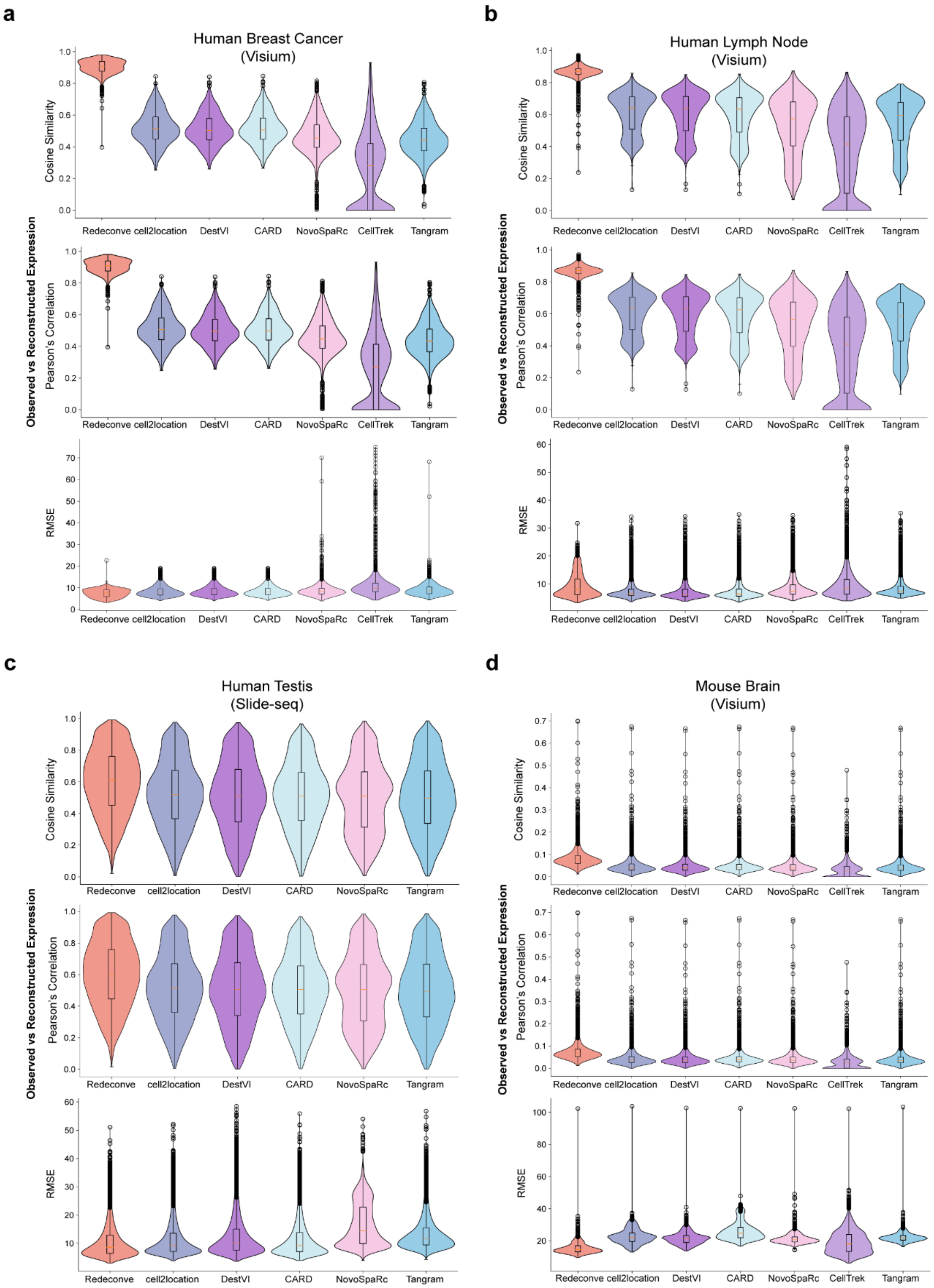
a
Human Breast Cancer (Visium)
b
Human Lymph Node (Visium)
c
Human Testis (Slide-seq)
d
Mouse Brain (Visium)
Observed vs Reconstructed Expression
Cosine Similarity
Pearson's Correlation
RMSE
Redeconve
cell2location
DestVI
CARD
NovoSpaRc
CellTrek
Tangram

Supplementary Fig 3. Violin and box plot of cosine similarities, Pearson's correlations and RMSEs between observed and reconstructed expression profiles on four datasets: a, Human Breast Cancer (n = 2426). b, Human Lymph Node (n = 4039). c, Human Testis (n = 36550). d, Mouse Brain (n = 2987). The center line and the bounds of box refer to median, Q1 and Q3 of scores and the whisker equal to 1.5*(Q3-Q1). The minimum and maximum scores refer to Q1-whisker and Q3+whisker. RMSE, Root-mean-square deviation. Source data are provided as a Source Data file.

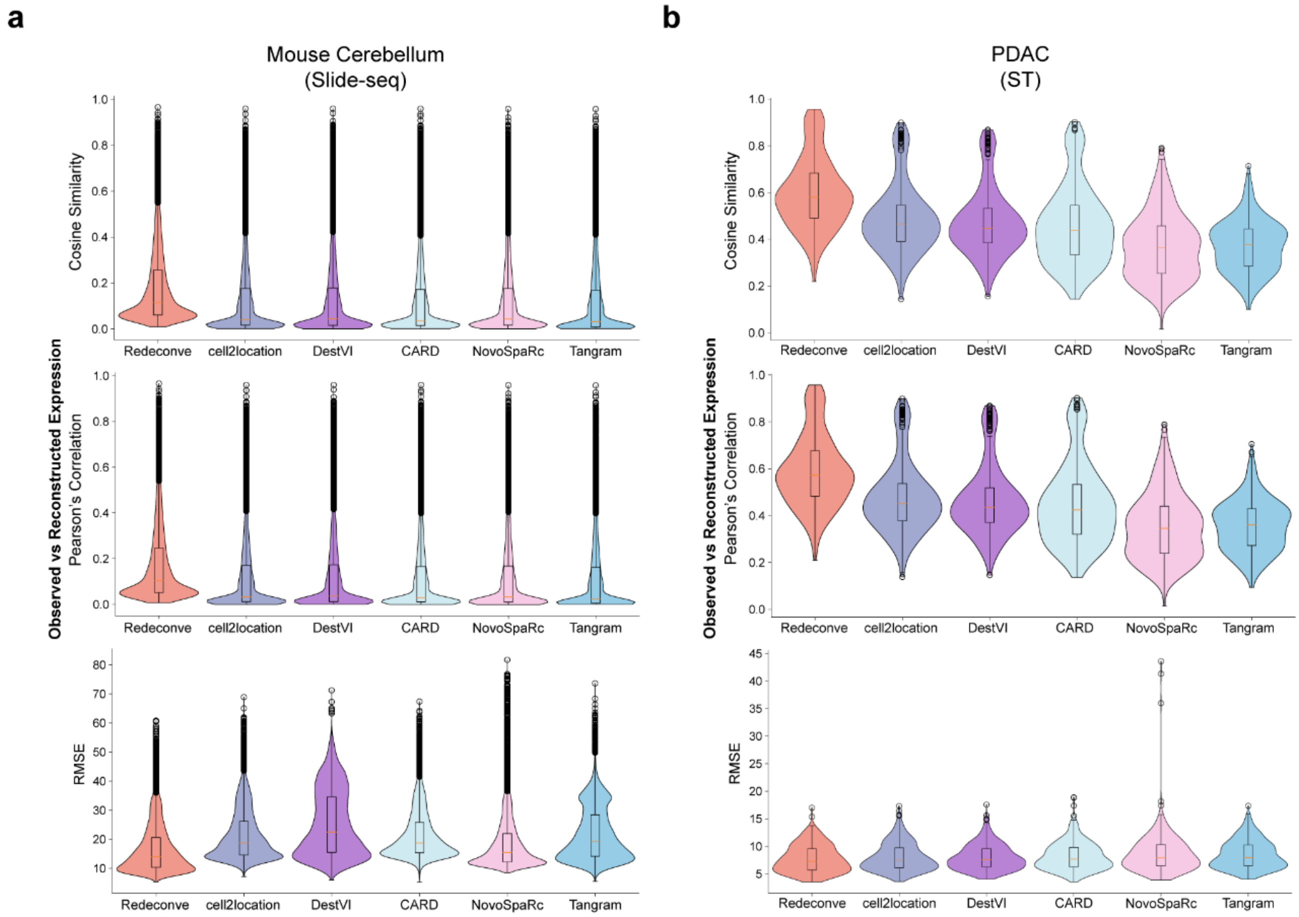

Supplementary Fig 4. Violin and box plot of cosine similarities, Pearson's correlations and RMSEs between observed and reconstructed expression profiles on two datasets: a, Mouse Cerebellum (n = 39431). b, PDAC (n = 428). The center line and the bounds of box refer to median, Q1 and Q3 of scores and the whisker equal to 1.5*(Q3-Q1). The minimum and maximum scores refer to Q1-whisker and Q3+whisker. RMSE, Root-mean-square deviation. Source data are provided as a Source Data file.

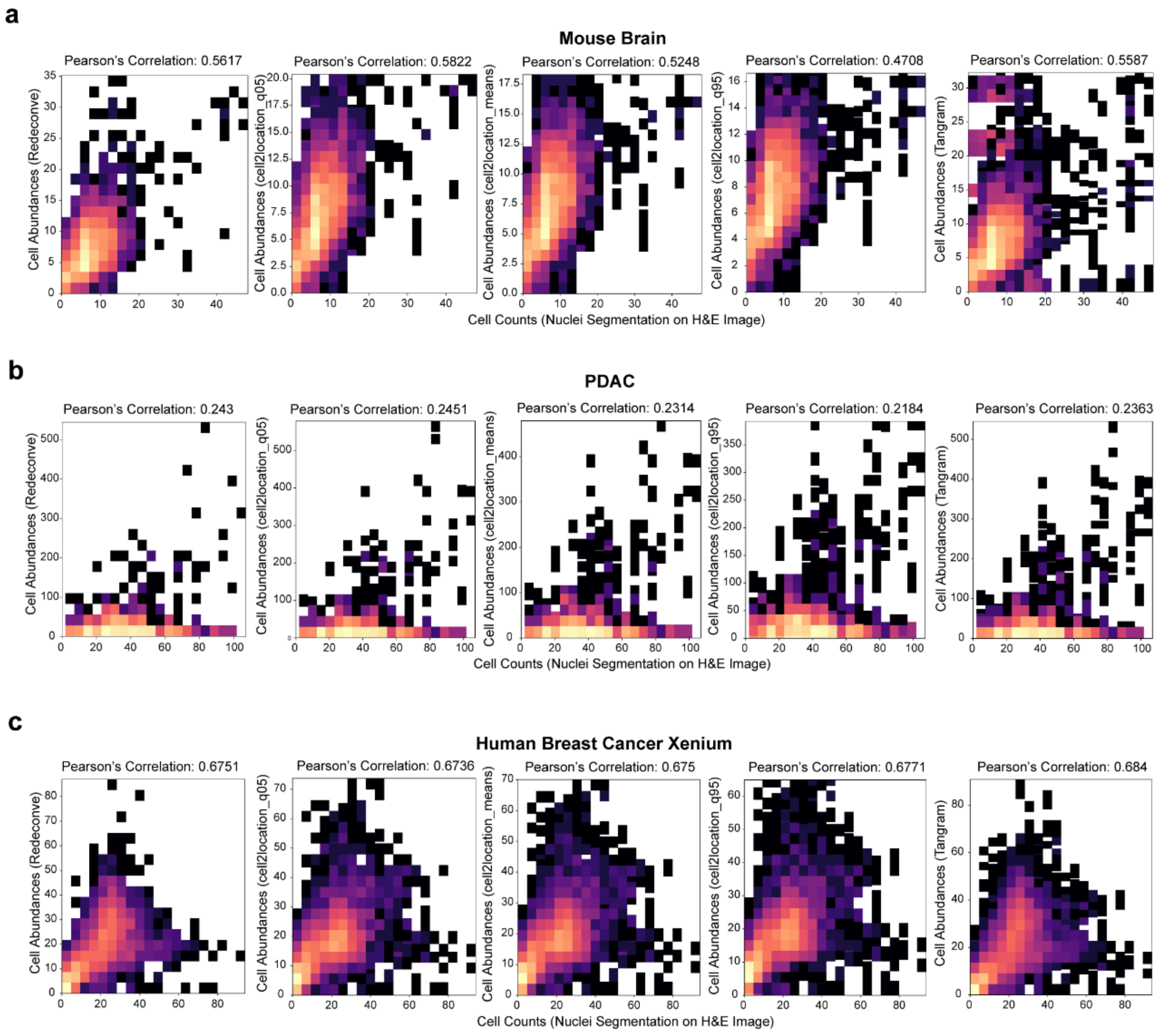

Supplementary Fig 5. Pearson's correlation of cell abundances estimated by different methods and ground truth (estimated by deep learning-based segmentation) for various datasets. a, Mouse Brain dataset. b, PDAC (pancreatic ductal adenocarcinoma) dataset. c, Human Breast Cancer Xenium dataset (scFFPE-seq reference). Source data are provided as a Source Data file.

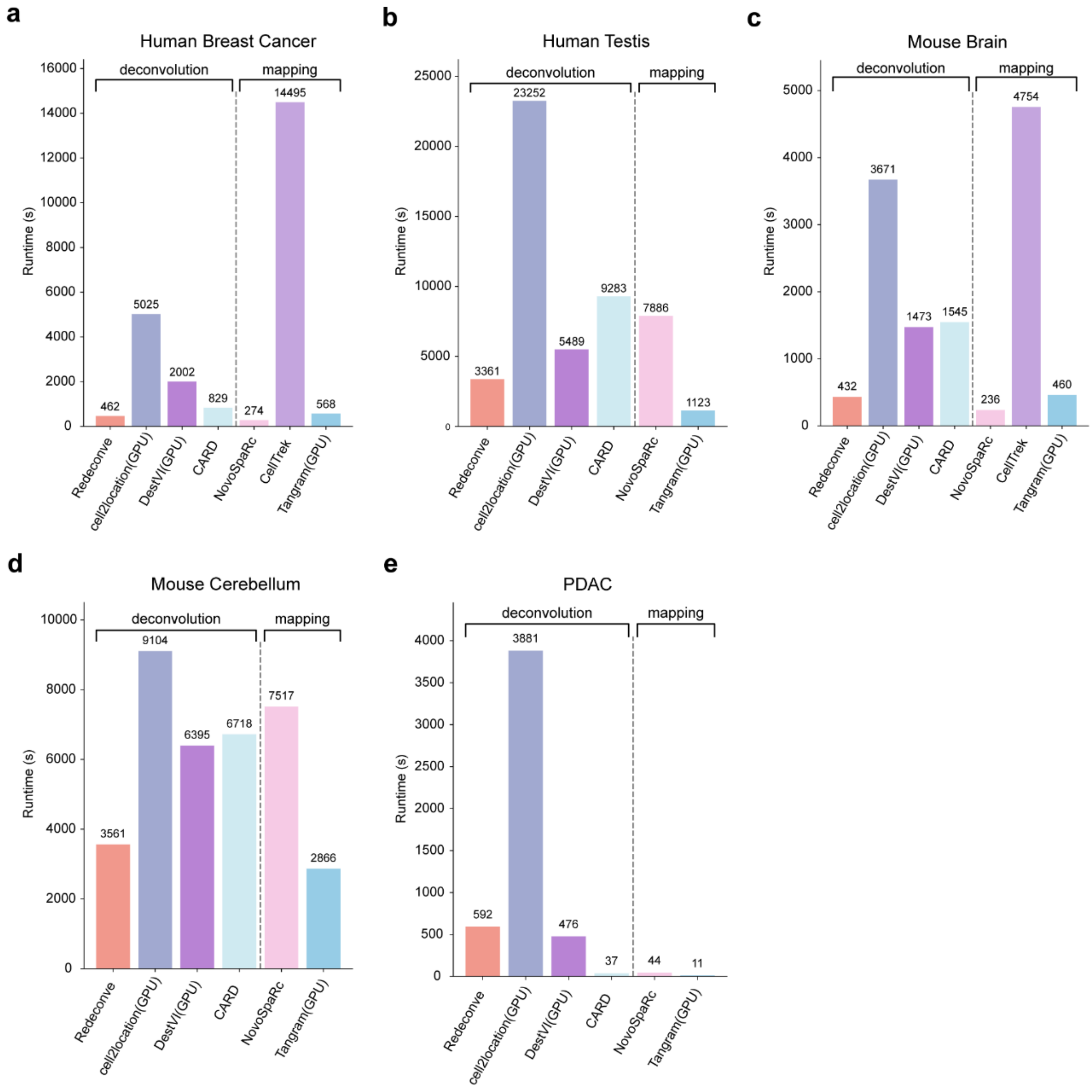

Supplementary Fig 6. Computational efficiency of different deconvolution-based and mapping-based algorithms on five datasets. a, Human Breast Cancer. b, Human Testis. c, Mouse Brain. d, Mouse Cerebellum. e, PDAC (pancreatic ductal adenocarcinoma).

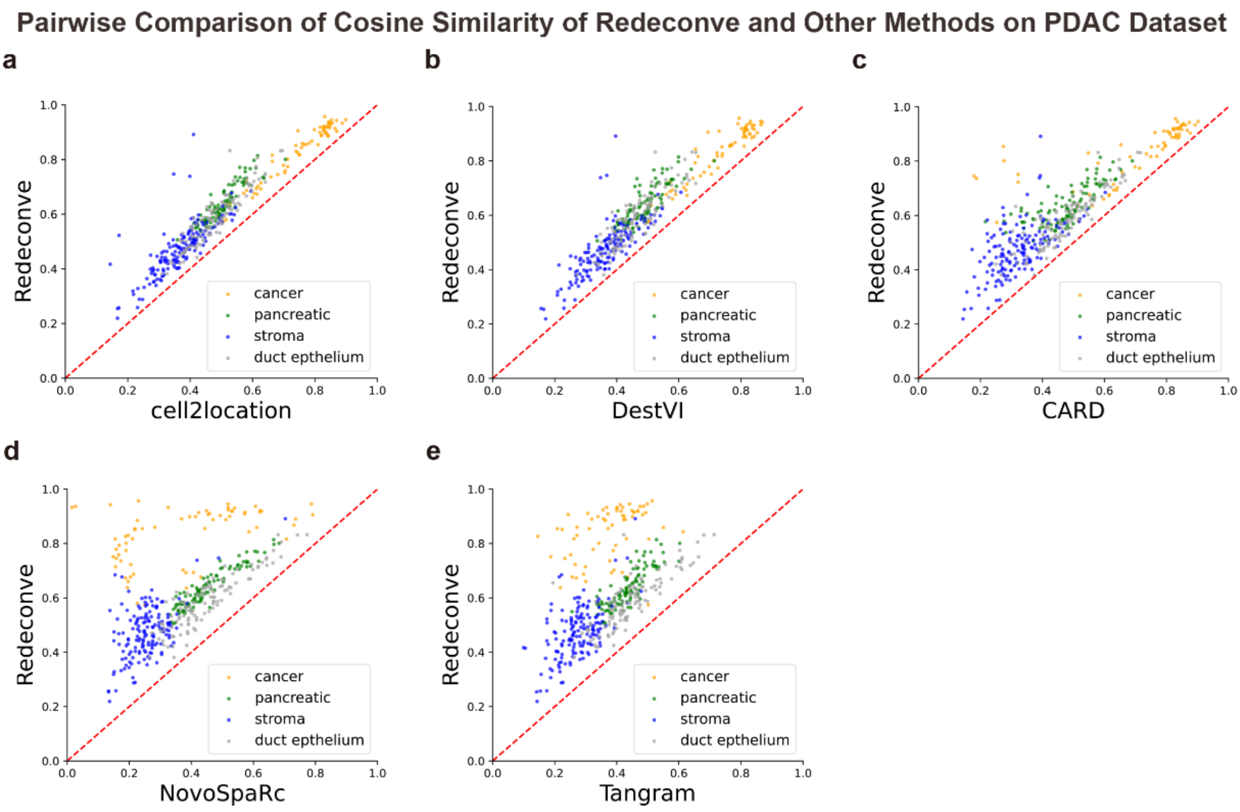

Supplementary Fig 7. Pairwise comparison between Redeconve and other methods regarding the spot-level reconstruction accuracy on PDAC dataset. a, Redeconve vs cell2location. b, Redeconve vs DestVI. c, Redeconve vs CARD. d, Redeconve vs NovoSpaRc. e, Redeconve vs Tangram. N = 428 spots. PDAC, pancreatic ductal adenocarcinoma. Source data are provided as a Source Data file.

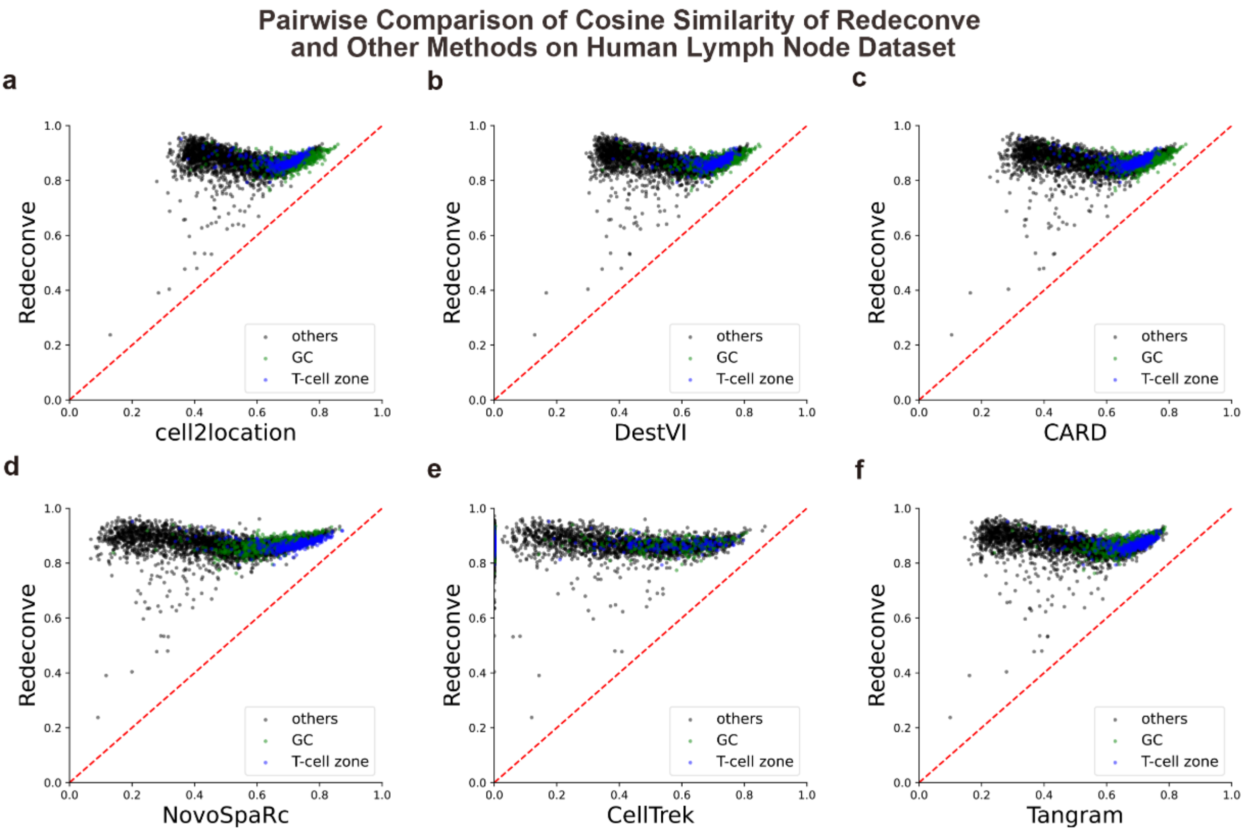

Supplementary Fig 8. Pairwise comparison between Redeconve and other methods regarding the spot-level reconstruction accuracy on human lymph nodes dataset. a, Redeconve vs cell2location. b, Redeconve vs DestVI. c, Redeconve vs CARD. d, Redeconve vs NovoSpaRc. e, Redeconve vs CellTrek. F, Redeconve vs Tangram. N = 4039 spots. Source data are provided as a Source Data file.

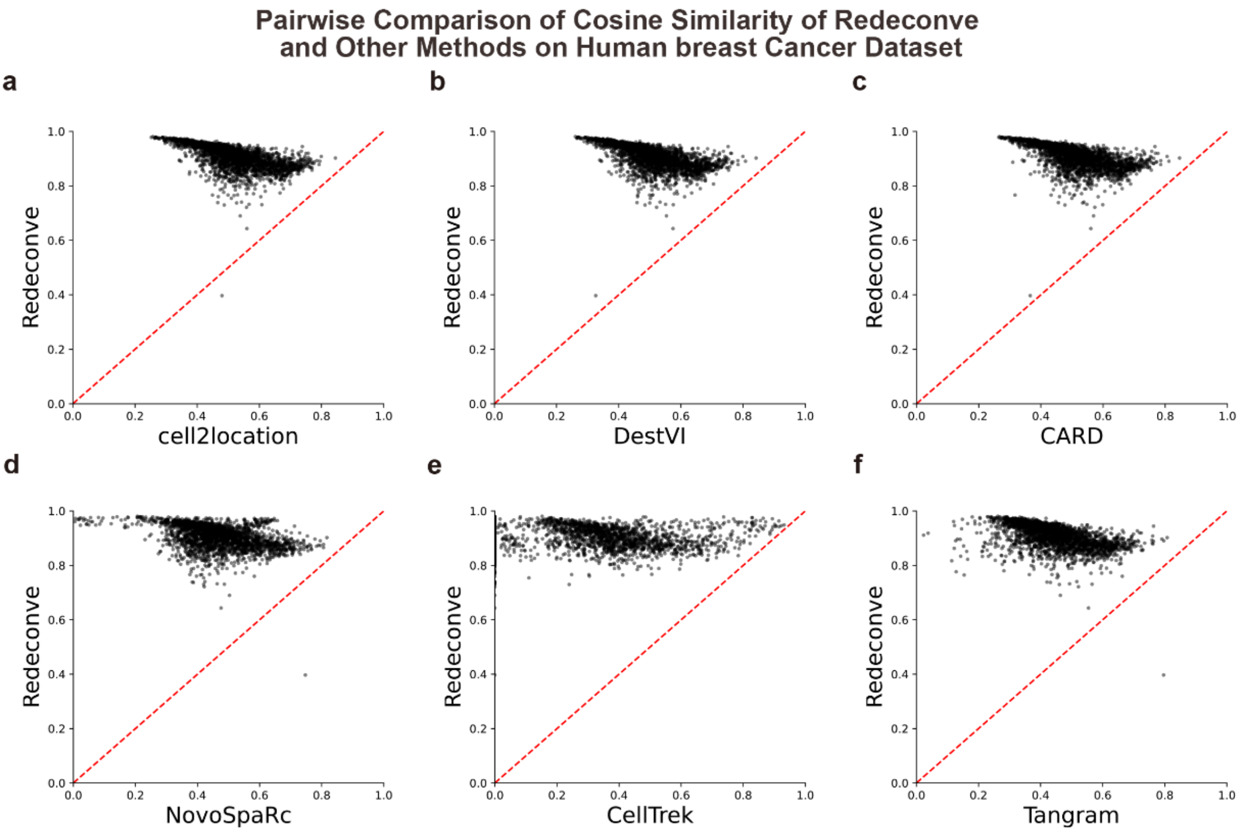

Supplementary Fig 9. Pairwise comparison between Redeconve and other methods regarding the spot-level reconstruction accuracy on human breast cancer dataset. a, Redeconve vs cell2location. b, Redeconve vs DestVI. c, Redeconve vs CARD. d, Redeconve vs NovoSpaRc. e, Redeconve vs CellTrek. f, Redeconve vs Tangram. N = 2426 spots. Source data are provided as a Source Data file.

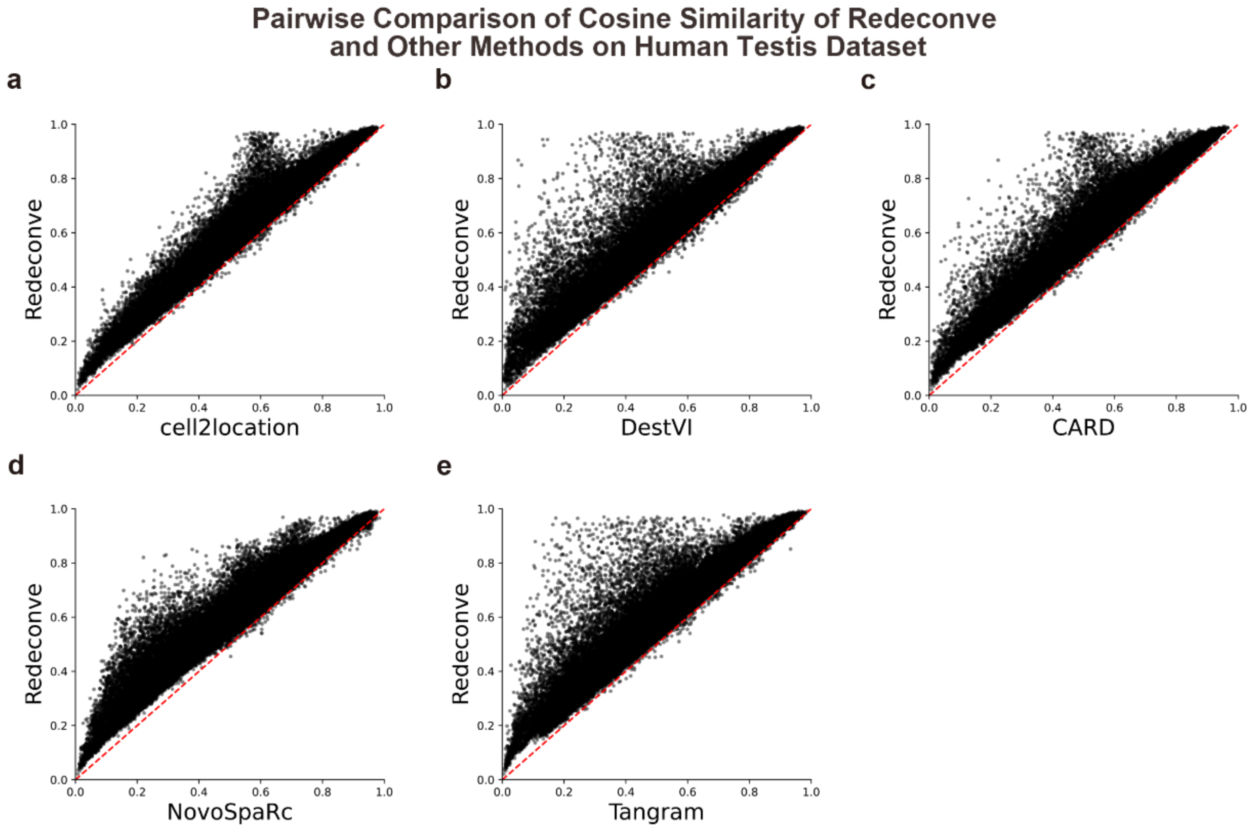

Supplementary Fig 10. Pairwise comparison between Redeconve and other methods regarding the spot-level reconstruction accuracy on human testis dataset. a, Redeconve vs cell2location. b, Redeconve vs DestVI. c, Redeconve vs CARD. d, Redeconve vs NovoSpaRc. e, Redeconve vs Tangram. N = 36550 spots. Source data are provided as a Source Data file.

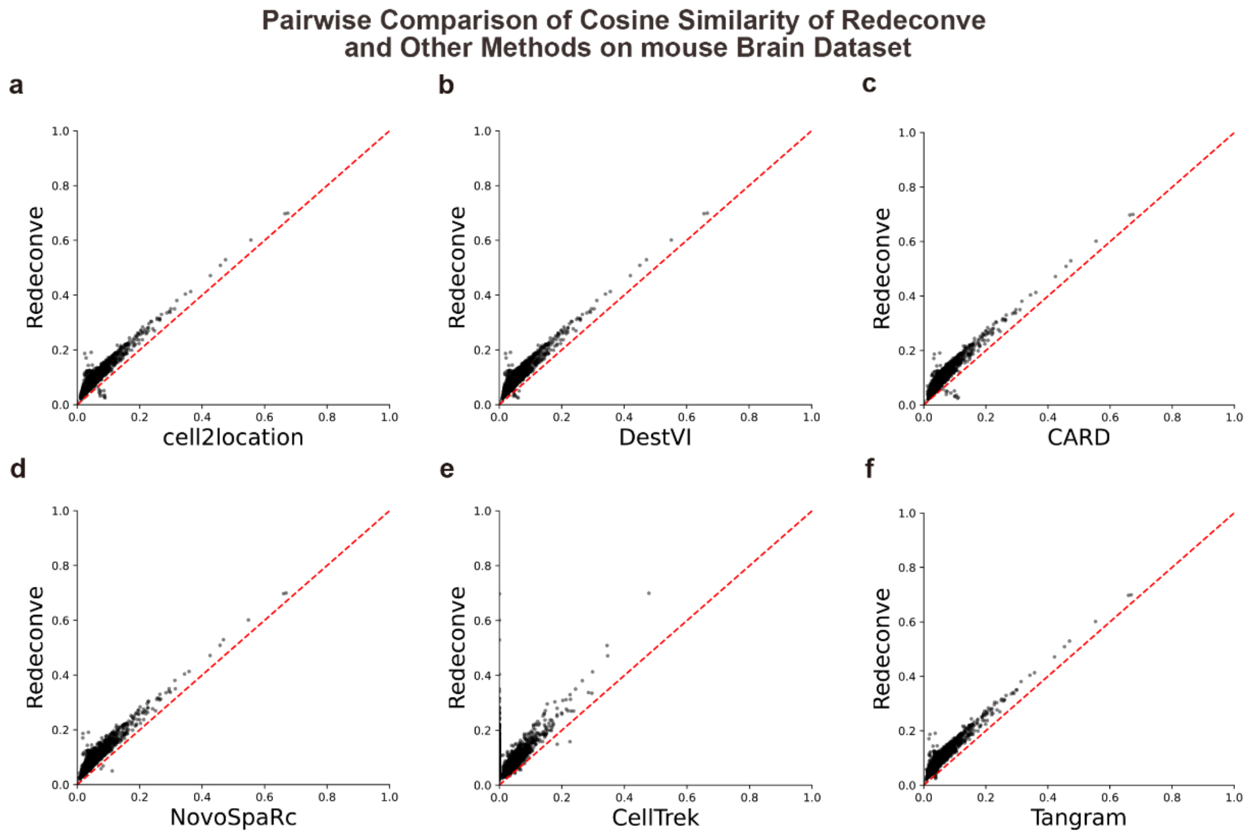

Supplementary Fig 11. Pairwise comparison between Redeconve and other methods regarding the spot-level reconstruction accuracy on mouse brain dataset. a, Redeconve vs cell2location. b, Redeconve vs DestVI. c, Redeconve vs CARD. d, Redeconve vs NovoSpaRc. e, Redeconve vs CellTrek. f, Redeconve vs Tangram. N = 2987 spots. Source data are provided as a Source Data file.

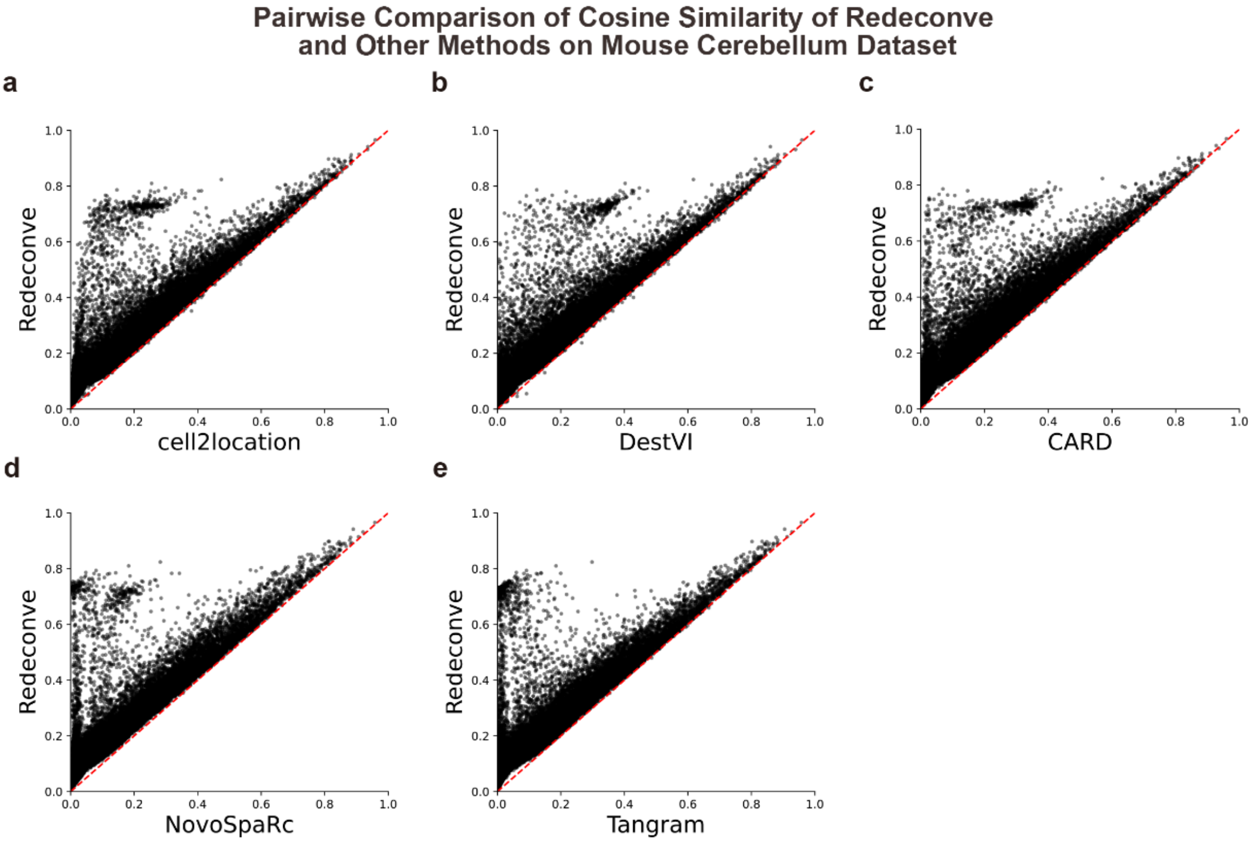

Supplementary Fig 12. Pairwise comparison between Redeconve and other methods regarding the spot-level reconstruction accuracy on mouse cerebellum dataset. a, Redeconve vs cell2location. b, Redeconve vs DestVI. c, Redeconve vs CARD. d, Redeconve vs NovoSpaRc. e, Redeconve vs Tangram. N = 39431 spots. Source data are provided as a Source Data file.

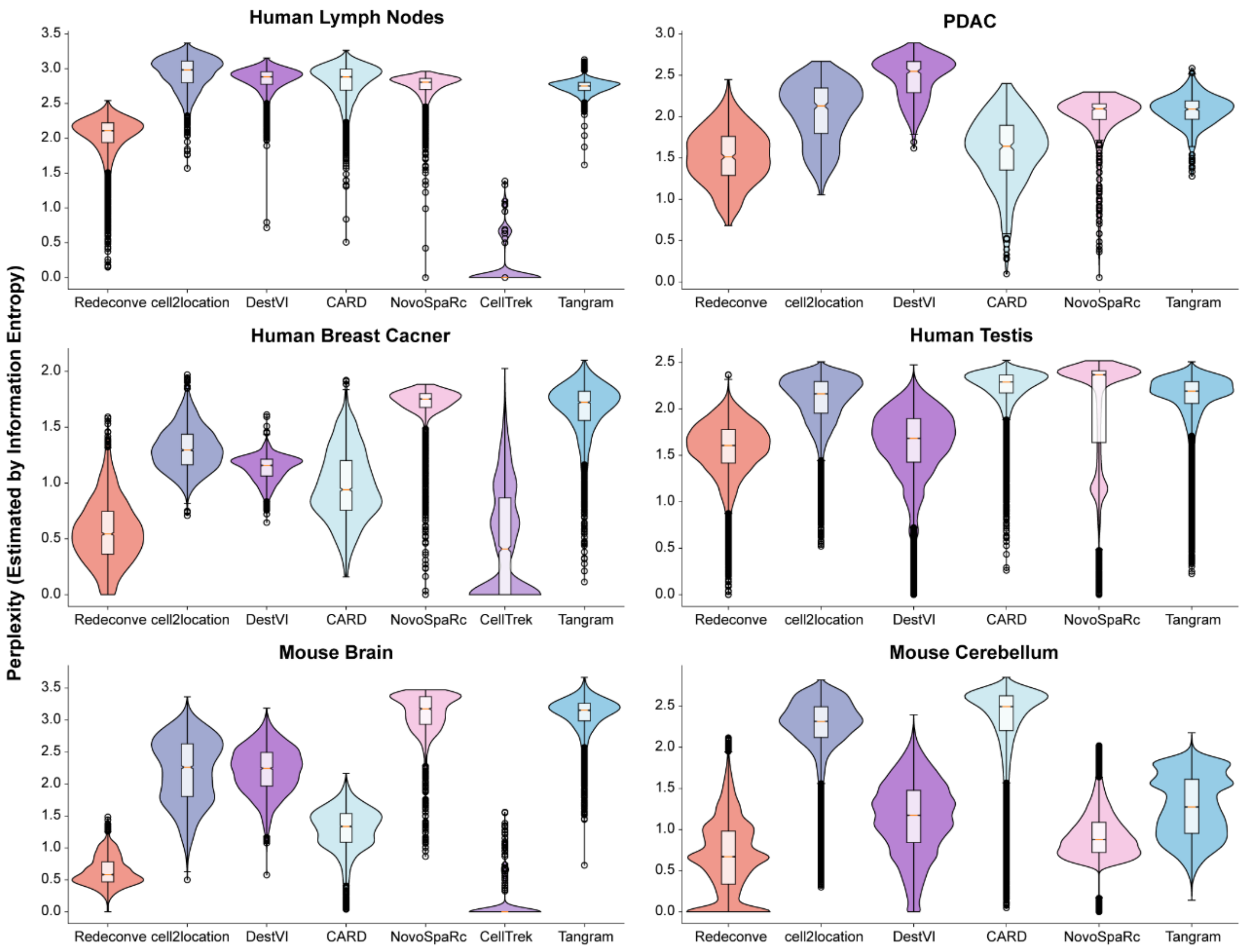

Supplementary Fig 13. Box and violin plots of spot-level information entropy among all algorithms on the six datasets. At specific spots, higher entropy indicates more complex of cellular composition. Redeconve generated results of which the cellular complexity was similar to the cell numbers per spot of different ST platforms. The center line and the bounds of box refer to median, Q1 and Q3 of scores and the whisker equal to 1.5*(Q3-Q1). The minimum and maximum scores refer to Q1-whisker and Q3+whisker. PDAC, pancreatic ductal adenocarcinoma. Source data are provided as a Source Data file.

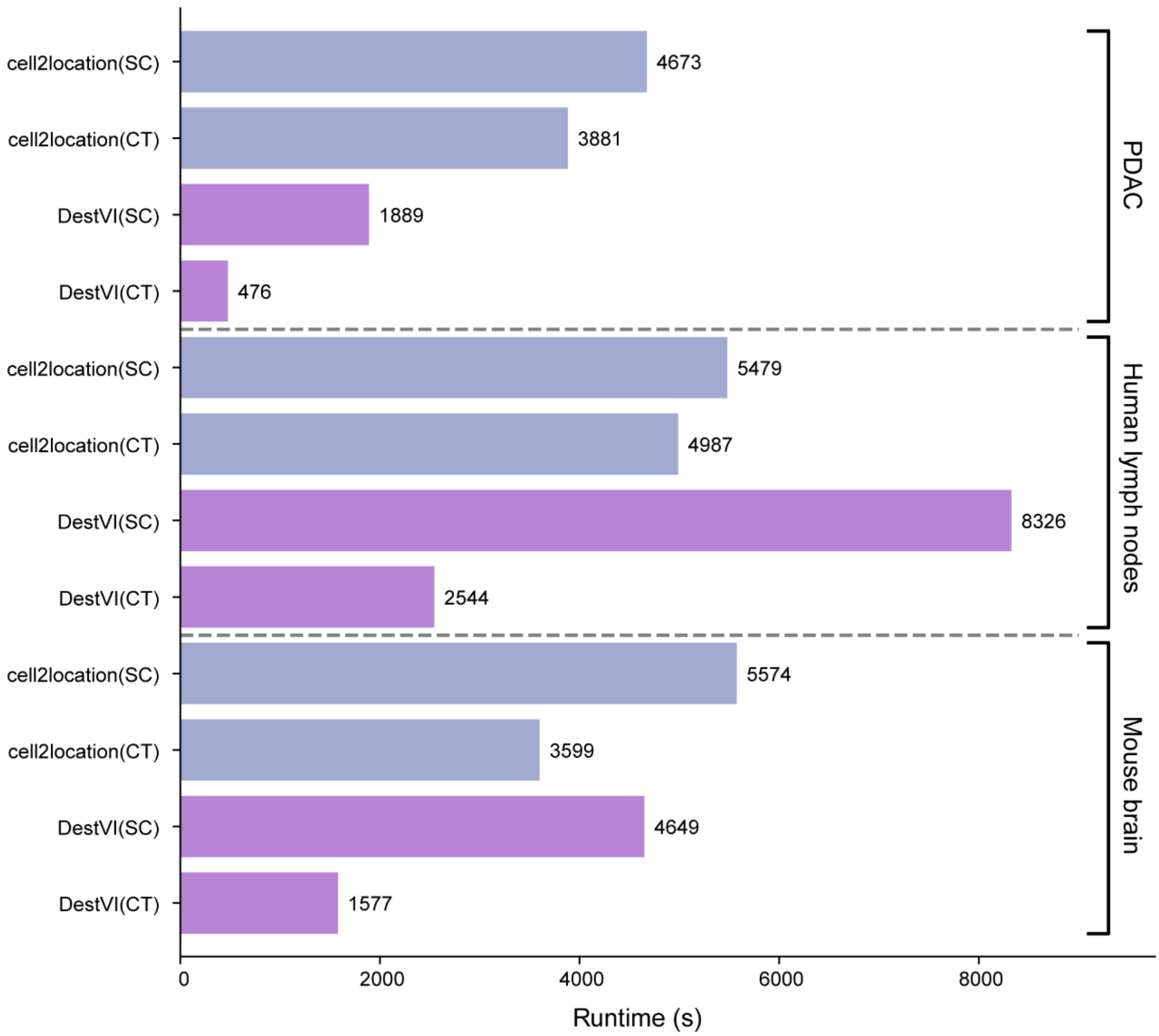

Supplementary Fig 14. Computational cost of cell2location and DestVI when 1000 single cells or cell type references served as inputs to perform deconvolution on the PDAC, human lymph nodes and mouse brain datasets. SC: 1000 single cells severd as inputs to approach single-cell resolution of deconvolution. CT: cell type references served as inputs for deconvolution. This test was performed on a single NVIDIA A40 card. PDAC, pancreatic ductal adenocarcinoma. SC, single-cell. CT, cell type.

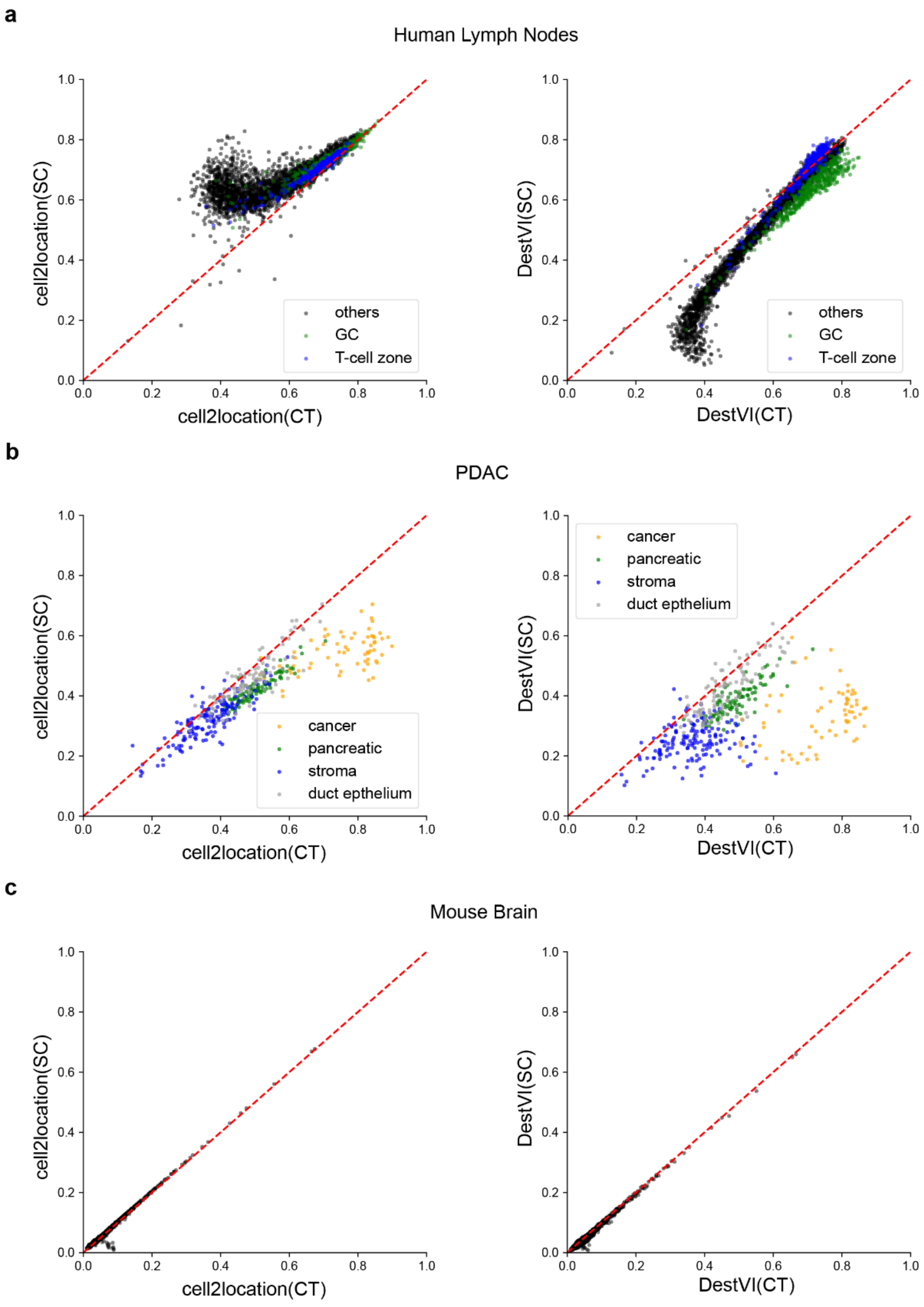
a
Human Lymph Nodes
cell2location(SC)
cell2location(CT)
others
GC
T-cell zone
DestVI(SC)
DestVI(CT)
b
PDAC
cancer
pancreatic
stroma
duct epthelium
c
Mouse Brain
0.0
0.2
0.4
0.6
0.8
1.0

Supplementary Fig 15. Pairwise comparison of spot-level reconstruction accuracy between single-cell (SC) and cell-type (CT) input in cell2location and DestVI on three datasets. a, the human lymph nodes dataset. b, the PDAC dataset. c, the mouse brain dataset. PDAC, pancreatic ductal adenocarcinoma. Source data are provided as a Source Data file.

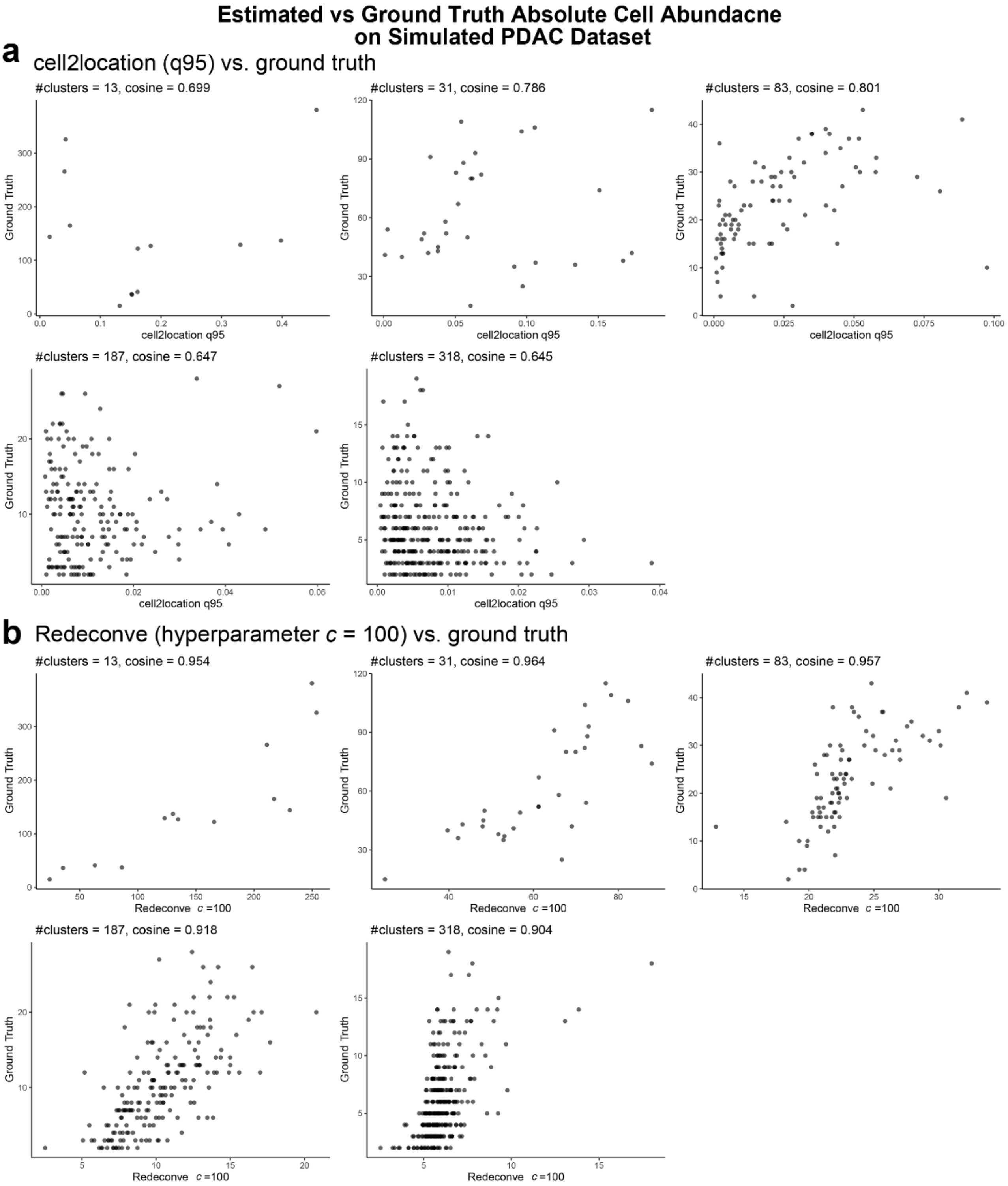

Supplementary Fig 16. Estimated cell abundance by cell2location and Redeconve with different cluster numbers vs. ground truth on simulated dataset 1 (PDAC). a, cell2location. b, Redeconve. PDAC, pancreatic ductal adenocarcinoma. Source data are provided as a Source Data file.

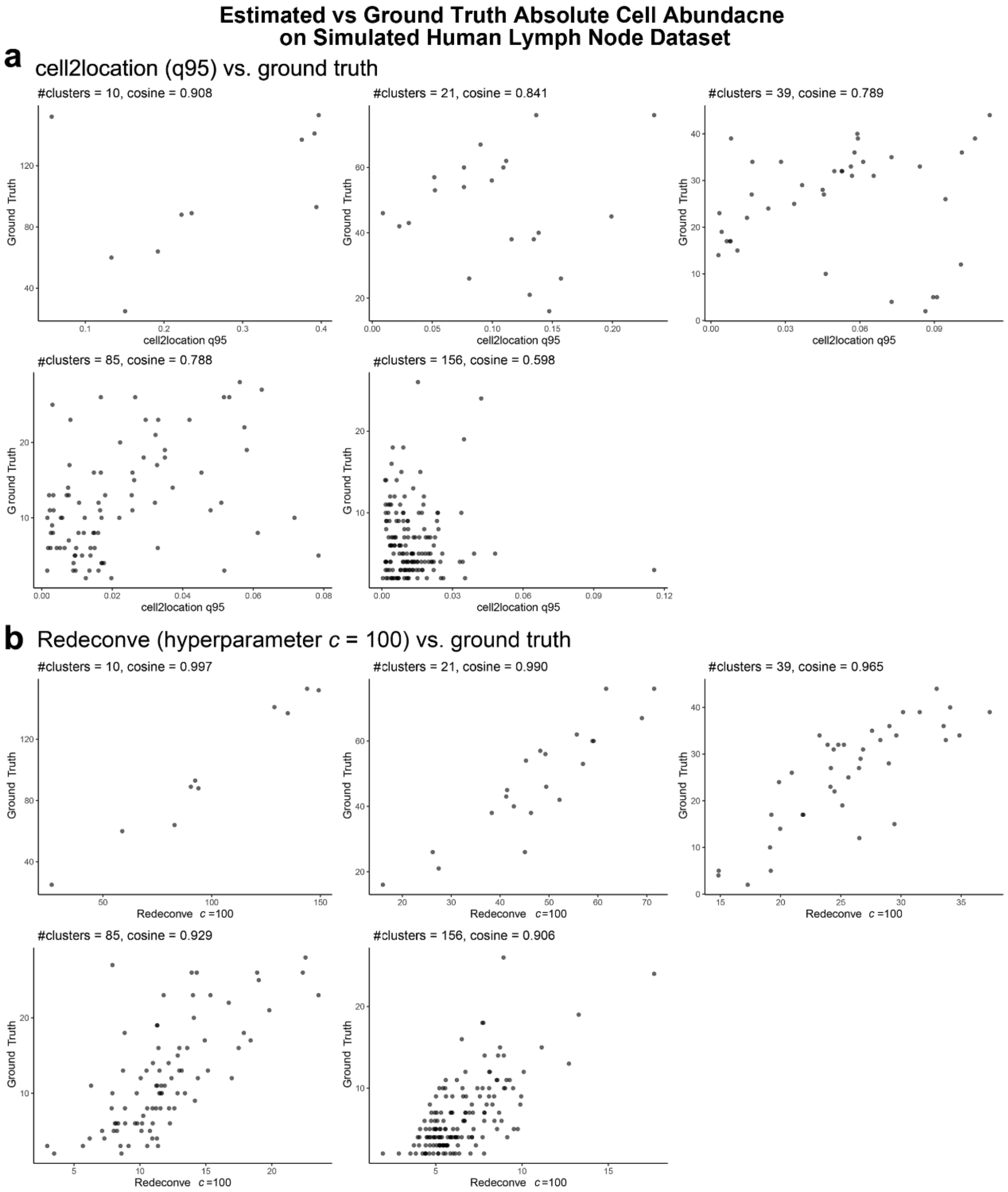

Supplementary Fig 17. Estimated cell abundance by cell2location and Redeconve with different cluster numbers vs. ground truth on simulated dataset 2 (human lymph node). a, cell2location. b, Redeconve. Source data are provided as a Source Data file.

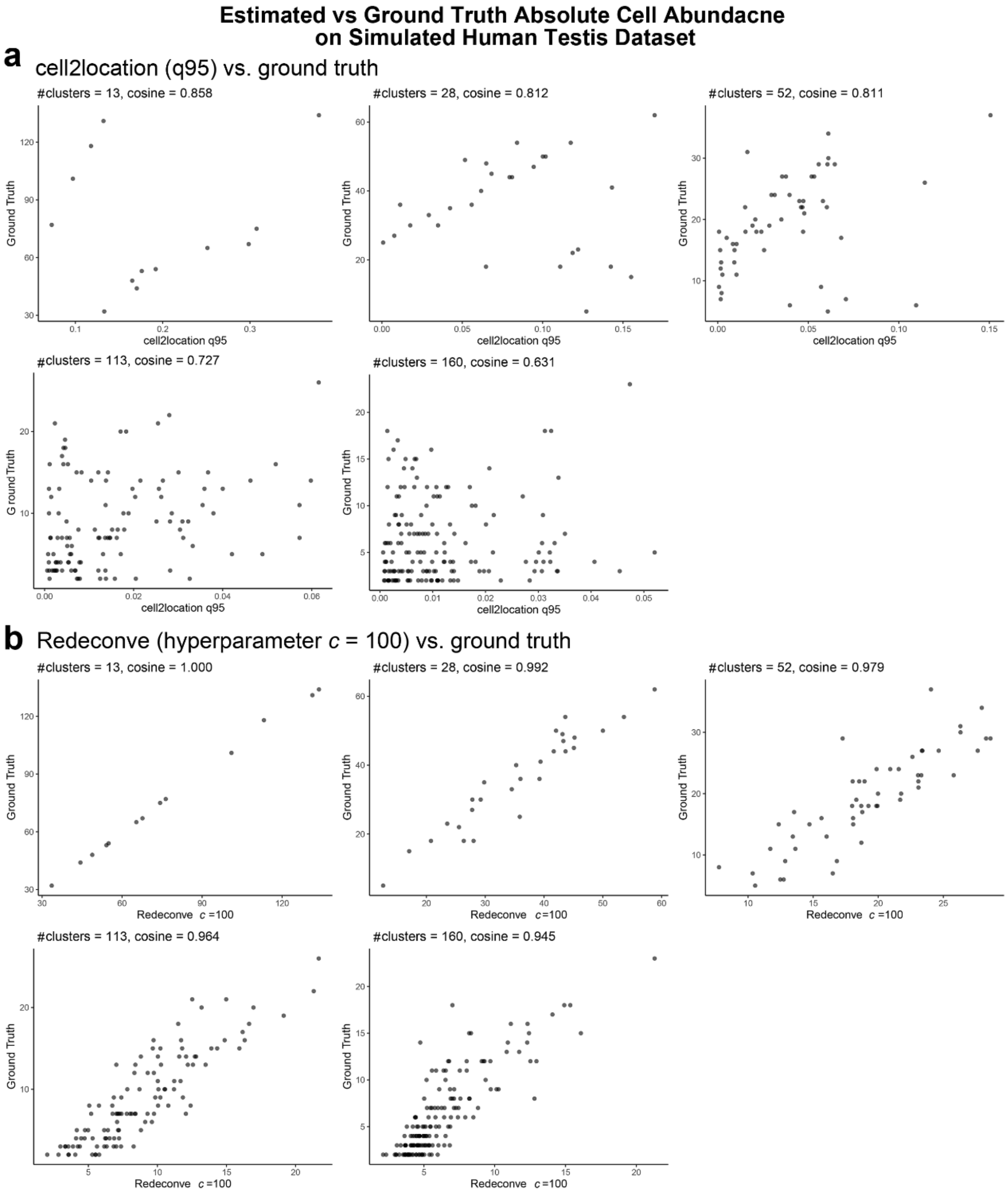

Supplementary Fig 18. Estimated cell abundance by cell2location and Redeconve with different cluster numbers vs. ground truth on simulated dataset 3 (human testis). a, cell2location. b, Redeconve. Source data are provided as a Source Data file.

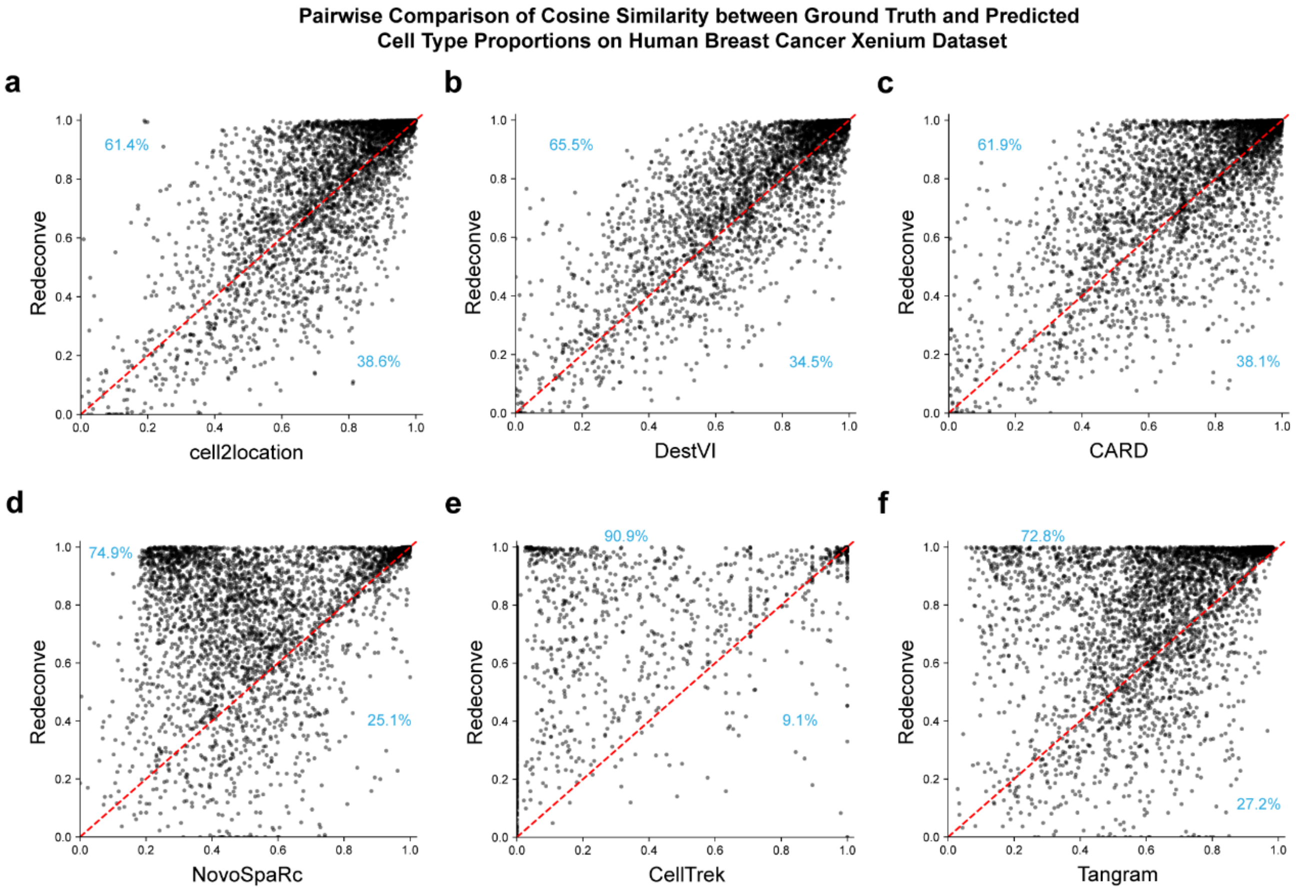

Supplementary Fig 19. Pairwise comparison between Redeconve and other methods regarding accuracy of cell type proportion estimation on the human breast cancer Xenium dataset. a, Redeconve vs cell2location. b, Redeconve vs DestVI. c, Redeconve vs CARD. d, Redeconve vs NovoSpaRc. e, Redeconve vs CellTrek. f, Redeconve vs Tangram. Each point refers to one spot. N = 3906 spots. Source data are provided as a Source Data file.

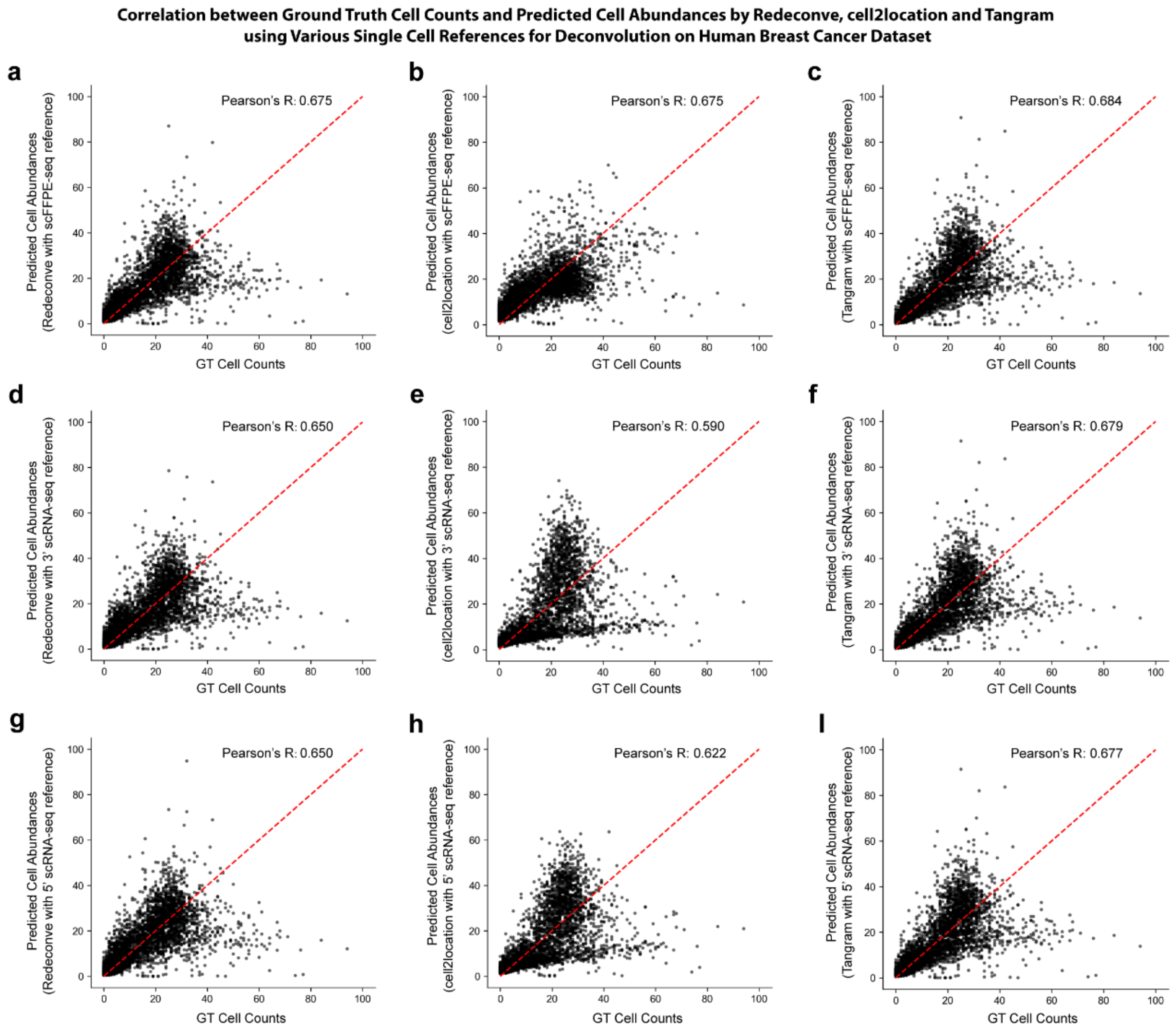

Supplementary Fig 20. Scatter plot of nuclei counts between GT (ground truth) and predictions from each model. The x axis representing GT (ground truth) nuclei counts, and y axis representing predicted nuclei counts for alternative methods. a, Redeconve with scFFPE-seq reference; b, cell2location with scFFPE-seq reference; c, Tangram with scFFPE-seq reference; d, Redeconve with 3' scRNA-seq reference; e, cell2location with 3' scRNA-seq reference; f, Tangram with 3' scRNA-seq reference; g, Redeconve with 5' scRNA-seq reference; h, cell2location with 5' scRNA-seq reference; i, Tangram with 5' scRNA-seq reference. Source data are provided as a Source Data file.

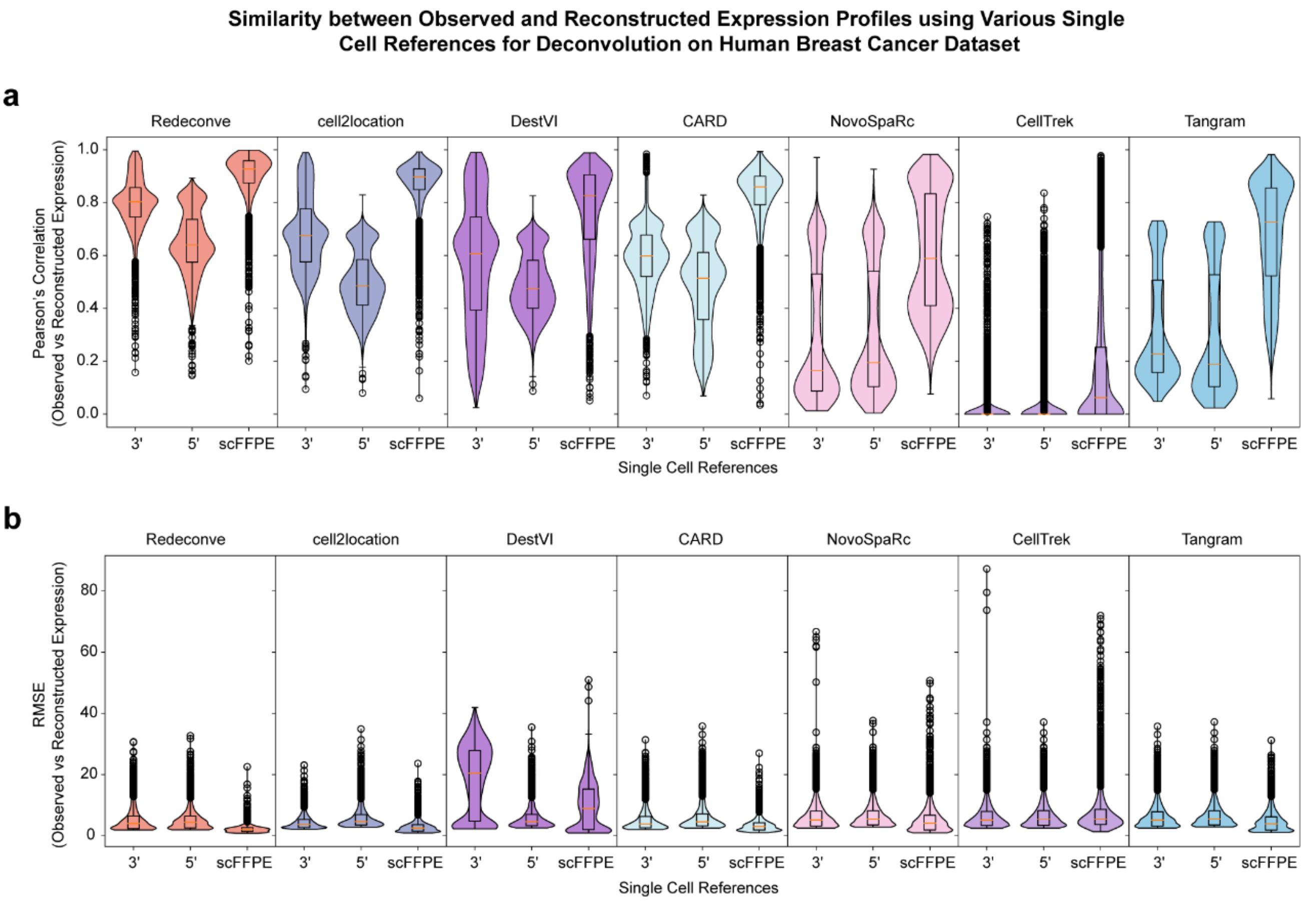

Supplementary Fig 21. Violin and box plot of Pearson's correlations and RMSEs between observed and reconstructed expression profiles for Redeconve and alternative approaches with different single cell references. a, result on Pearson's correlations. b, result on RMSEs. The center line and the bounds of box refer to median, Q1 and Q3 of scores and the whisker equal to 1.5*(Q3-Q1). The minimum and maximum scores refer to Q1-whisker and Q3+whisker. Source data are provided as a Source Data file. RMSE, Root-mean-square deviation. Source data are provided as a Source Data file.

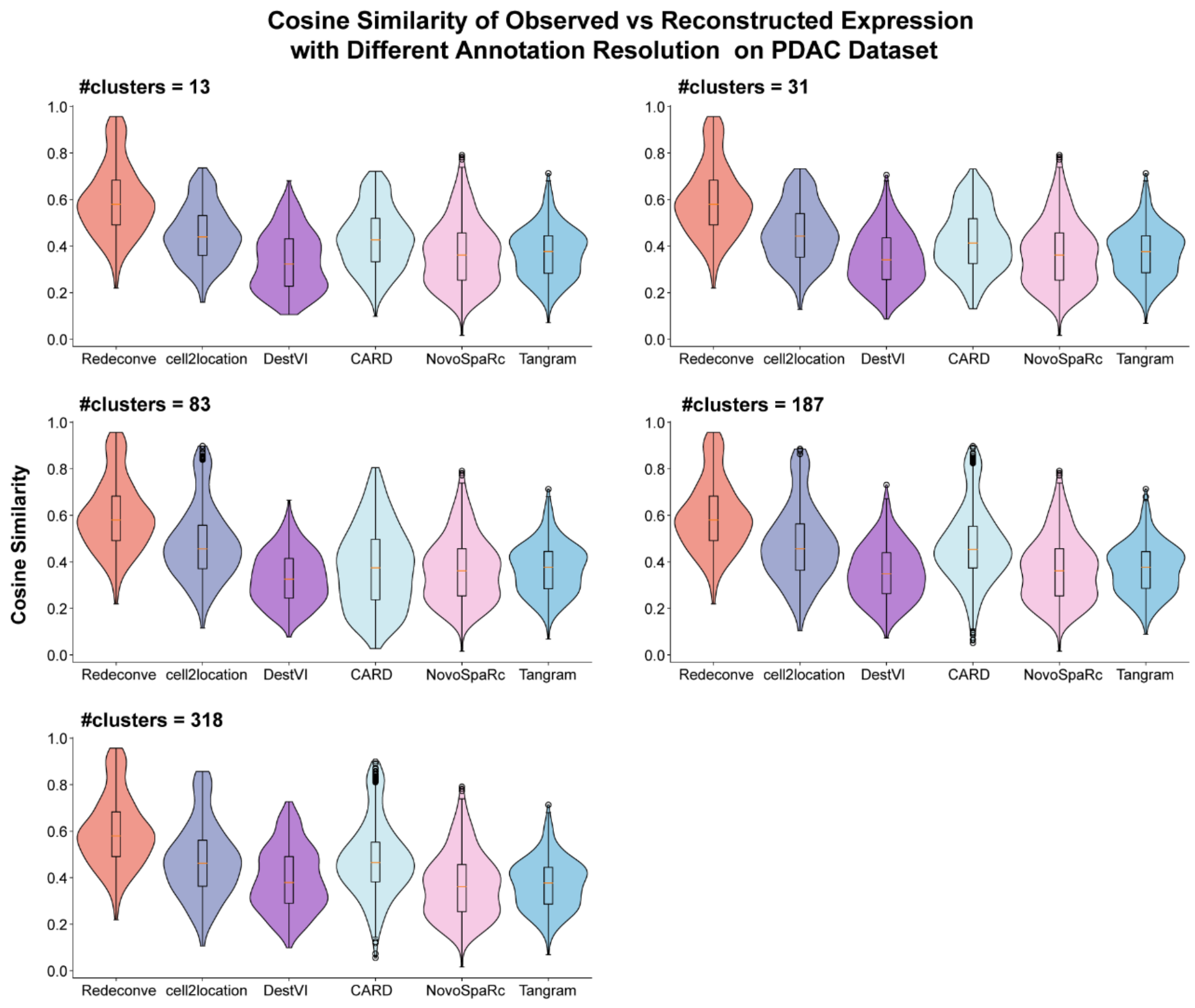

Supplementary Fig 22. Violin and box plot of cosine similarity with different annotation resolutions on PDAC dataset. Single cells were clustered with different resolutions by Seurat and used for deconvolution. The center line and the bounds of box refer to median, Q1 and Q3 of scores and the whisker equal to 1.5*(Q3-Q1). The minimum and maximum scores refer to Q1-whisker and Q3+whisker. Source data are provided as a Source Data file.

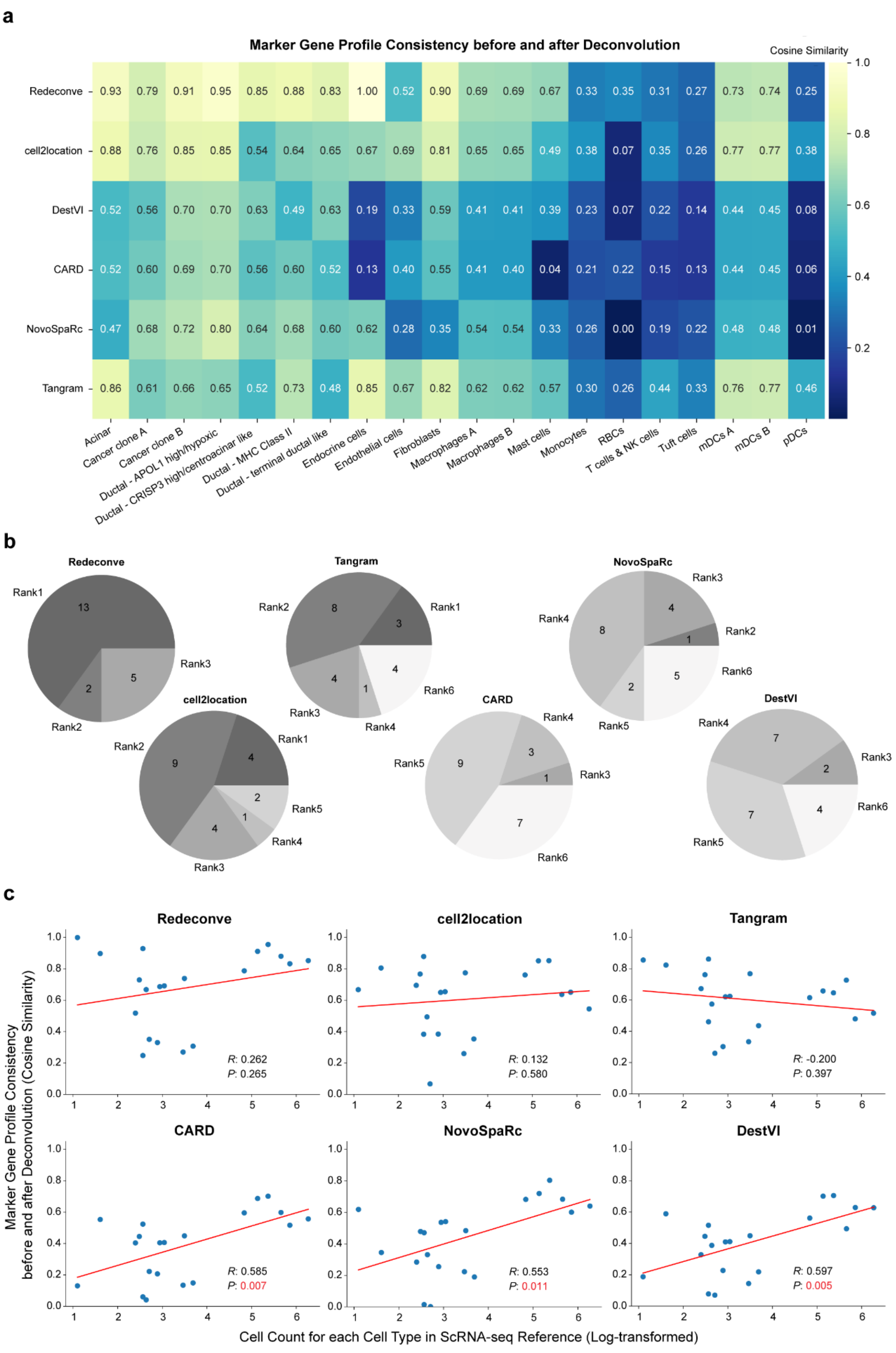
a
Marker Gene Profile Consistency before and after Deconvolution
Cosine Similarity
1.0
0.8
0.6
0.4
0.2
Redeconve 0.93 0.79 0.91 0.95 0.85 0.88 0.83 1.00 0.52 0.90 0.69 0.69 0.67 0.33 0.35 0.31 0.27 0.73 0.74 0.25
cell2location 0.88 0.76 0.85 0.85 0.54 0.64 0.65 0.67 0.69 0.81 0.65 0.65 0.49 0.38 0.07 0.35 0.26 0.77 0.77 0.38
DestVI 0.52 0.56 0.70 0.70 0.63 0.49 0.63 0.19 0.33 0.59 0.41 0.41 0.39 0.23 0.07 0.22 0.14 0.44 0.45 0.08
CARD 0.52 0.60 0.69 0.70 0.56 0.60 0.52 0.13 0.40 0.55 0.41 0.40 0.04 0.21 0.22 0.15 0.13 0.44 0.45 0.06
NovoSpaRc 0.47 0.68 0.72 0.80 0.64 0.68 0.60 0.62 0.28 0.35 0.54 0.54 0.33 0.26 0.00 0.19 0.22 0.48 0.48 0.01
Tangram 0.86 0.61 0.66 0.65 0.52 0.73 0.48 0.85 0.67 0.82 0.62 0.62 0.57 0.30 0.26 0.44 0.33 0.76 0.77 0.46
Acinar
Cancer clone A
Cancer clone B
Ductal - APOL1 high/hypoxic
Ductal - CRISP3 high/centroacinar like
Ductal - MHC Class II
Ductal - terminal ductal like
Endocrine cells
Endothelial cells
Fibroblasts
Macrophages A
Macrophages B
Mast cells
Monocytes
RBCs
T cells & NK cells
Tuft cells
mDCs A
mDCs B
pDCs
b
Redeconve
Rank1 13
Rank2 2
Rank3 5
Tangram
Rank1 3
Rank2 8
Rank3 4
Rank4 1
Rank6 4
NovoSpaRc
Rank2 1
Rank3 4
Rank4 8
Rank5 2
Rank6 5
cell2location
Rank1 4
Rank2 9
Rank3 4
Rank4 1
Rank5 2
CARD
Rank3 1
Rank4 3
Rank5 9
Rank6 7
DestVI
Rank3 2
Rank4 7
Rank5 7
Rank6 4
c
Redeconve R: 0.262 P: 0.265
cell2location R: 0.132 P: 0.580
Tangram R: -0.200 P: 0.397
CARD R: 0.585 P: 0.007
NovoSpaRc R: 0.553 P: 0.011
DestVI R: 0.597 P: 0.005
Marker Gene Profile Consistency before and after Deconvolution (Cosine Similarity)
Cell Count for each Cell Type in ScRNA-seq Reference (Log-transformed)

Supplementary Fig 23. Benchmarking Redeconve and alternative methods at cell type level based on the PDAC dataset. a, heatmap of cosine similarities of marker genes expression between ST observation and reconstructed profiles by Redeconve and alternative methods. b, Pie charts demonstrated the algorithm ranks of individual cell types in a. c, Scatter plot of log-transformed cell counts in scRNA-seq data versus expression similarities at the cell type level in a. N = 20 cell types. PDAC, pancreatic ductal adenocarcinoma. Source data are provided as a Source Data file.

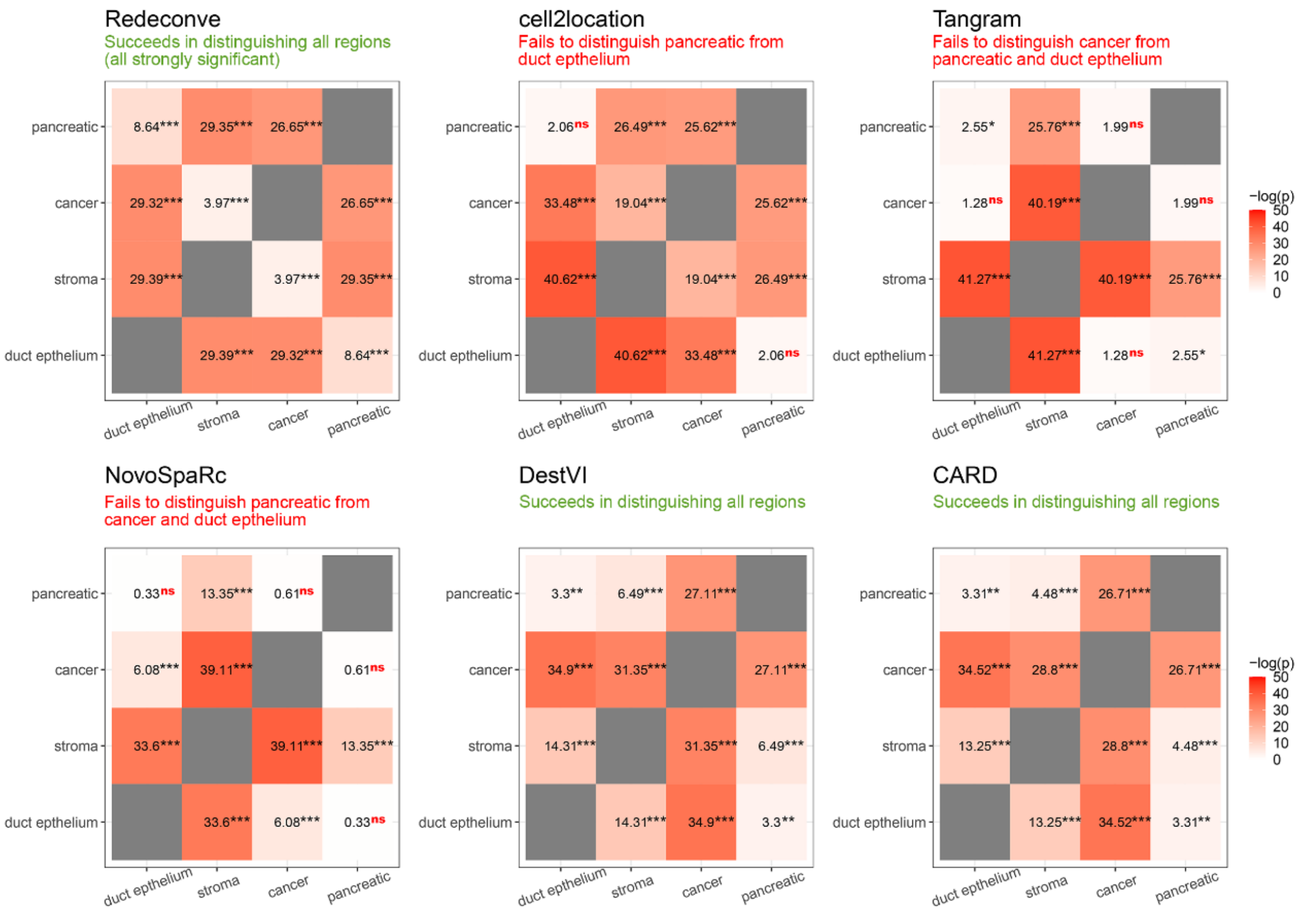

Supplementary Fig 24. Heatmap of Wilcoxon rank sum test for PDAC (pancreatic ductal adenocarcinoma) dataset. PCA (principle component analysis) was first performed on the cell-type result of each method. PC1 was then used for two-sided Wilcoxon rank sum test. Cell2location, Tangram and NovoSpaRc all generated non-significant region pairs (p-value > 0.008, i.e. Bonferroni corrected p-value threshold 0.05/6). All region pairs of Redeconve are strongly significant (p-value < 0.001/6 = 0.002). Meaning of symbols: ns: non-significant, p > 0.05/6 = 0.008, -log(p) < 2.08; *: 0.01/6 = 0.002 < p ⩽ 0.008, 2.08 < -log(p) < 2.78; **: 0.001/6 = 0.0002 < p ⩽ 0.002, 2.78 < -log(p) < 3.78; ***: p < 0.0002, -log(p) > 3.78.

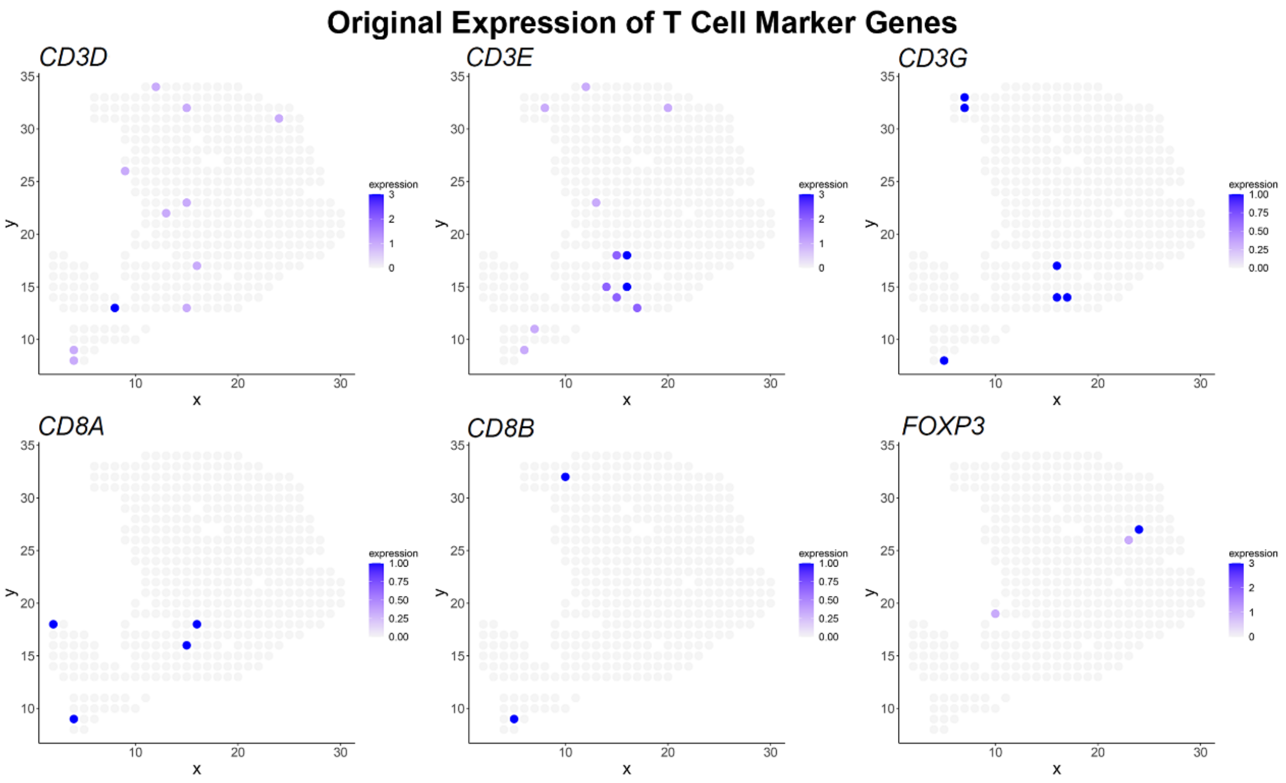

Supplementary Fig 25. Visualization of the spatial distribution of *CD3*, *CD8* and *FOXP3*. These genes showed localized patterns, consistent with the result of Redeconve that T cells are not evenly distributed. Source data are provided as a Source Data file.

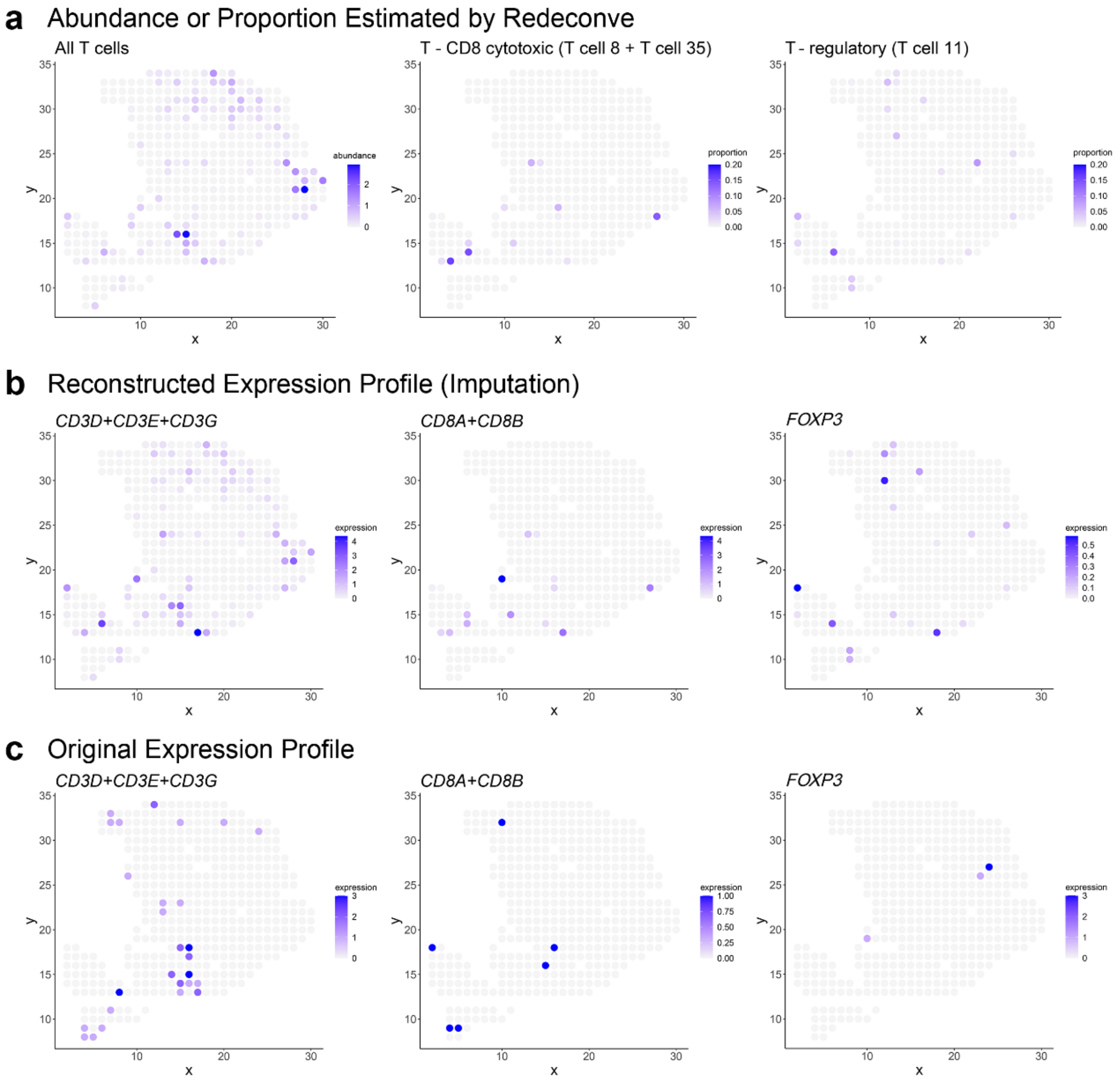

Supplementary Fig 26. Comparison of Redeconve-estimated T cell distribution, reconstructed and original expression of T-cell-related genes. a, estimated T cell abundance by Redeconve; b, Reconstructed expression (imputation) of *CD3*, *CD8* and *FOXP3*; c, original expression of *CD3*, *CD8* and *FOXP3*. Source data are provided as a Source Data file.

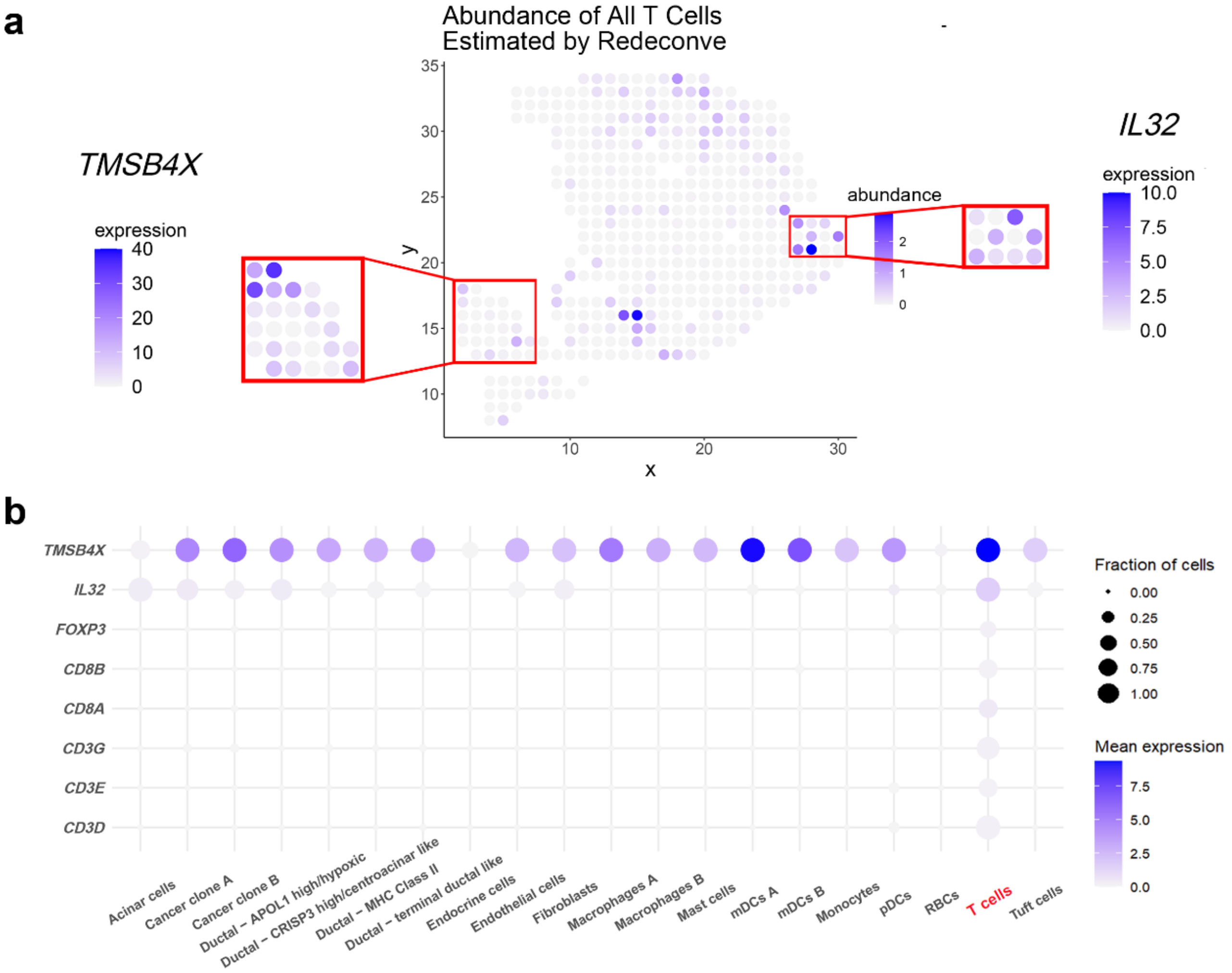

Supplementary Fig 27. Genes beyond *CD3* supporting existence of T cells. a, estimated T cell abundance by Redeconve and part of the expression of other T cell related genes *TMSB4X* and *IL32*; b, dot plot displaying specificity of *TMSB4X*, *IL32* and other T cell marker genes. Source data are provided as a Source Data file.

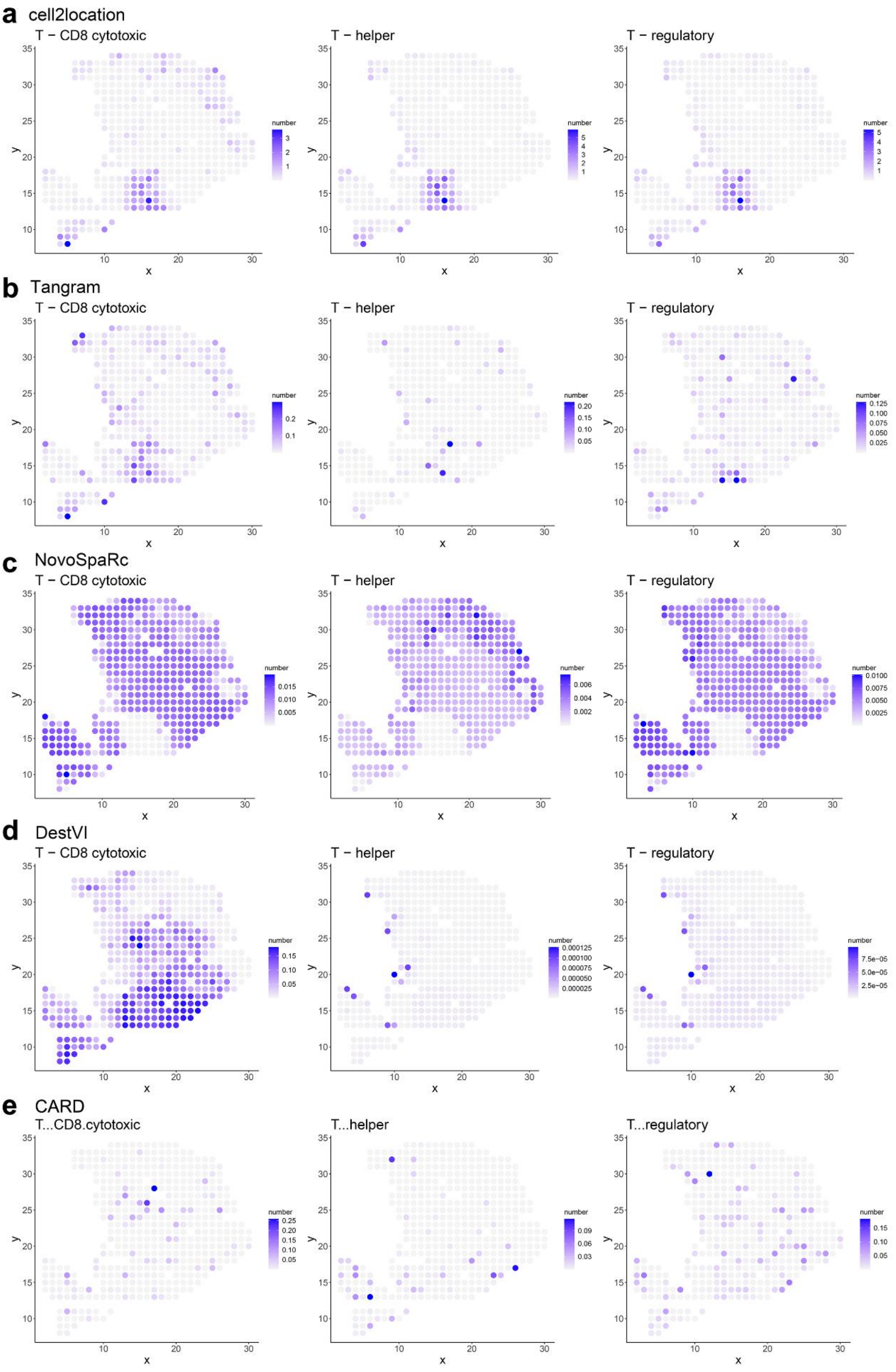
a cell2location
T − CD8 cytotoxic
T − helper
T − regulatory
b Tangram
T − CD8 cytotoxic
T − helper
T − regulatory
c NovoSpaRc
T − CD8 cytotoxic
T − helper
T − regulatory
d DestVI
T − CD8 cytotoxic
T − helper
T − regulatory
e CARD
T...CD8.cytotoxic
T...helper
T...regulatory
number
x
y

Supplementary Fig 28. Spatial distribution of three T cell subtypes by other methods. a, cell2location. b, Tangram. c, NovoSpaRc. d, DestVI. e, CARD. Source data are provided as a Source Data file.

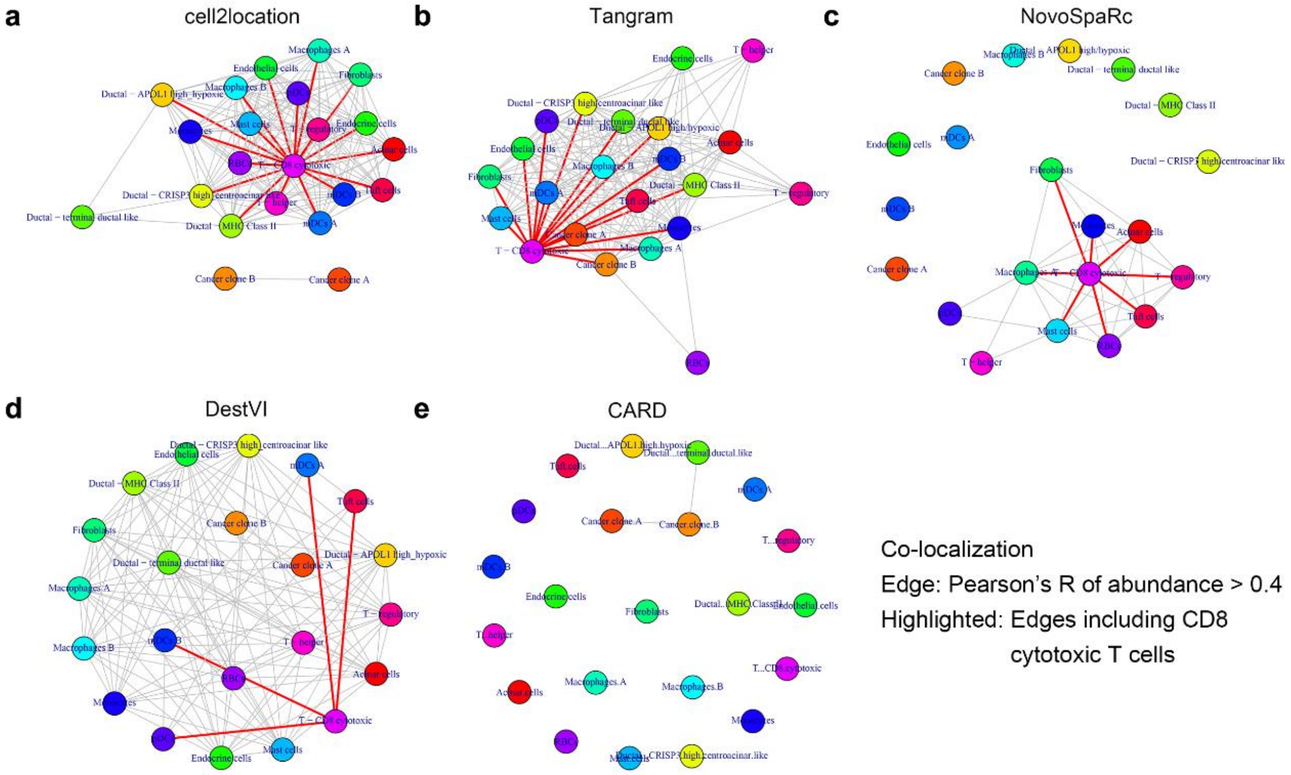

Supplementary Fig 29. Co-localization analysis of alternative methods with finer-annotated T cells. Adjacent matrix was defined by Pearson correlation coefficient (PCC) and the cut-off was set to 0.4, same as Redeconve. a, cell2location. b, Tangram. c, NovoSpaRc. d, DestVI. e, CARD. Source data are provided as a Source Data file.

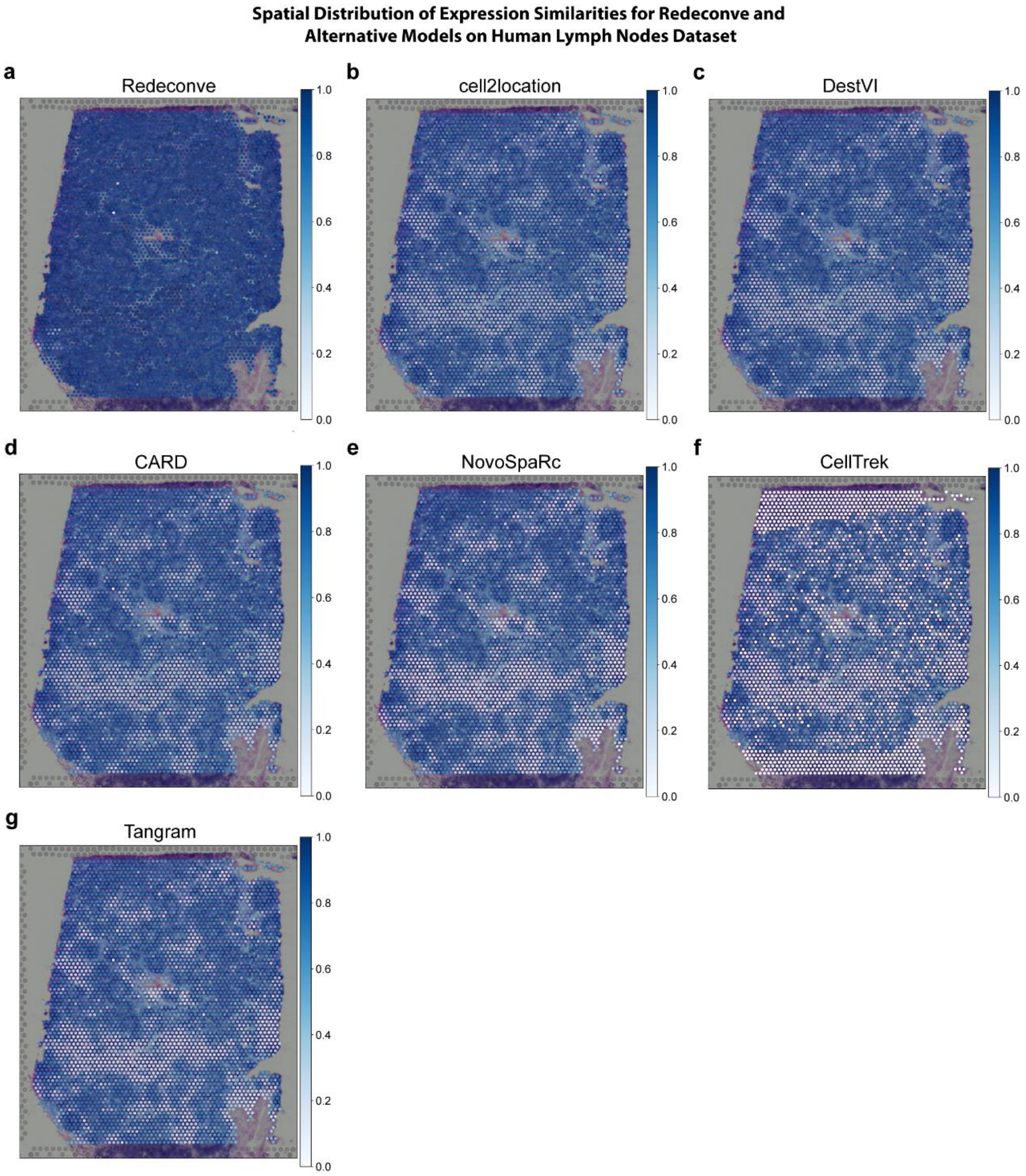

Supplementary Fig 30. Spatial distribution of reconstruction accuracy for spots among different algorithms on the human lymph nodes dataset. Color represents degree of cosine similarity. a, Redeconve. b, cell2location. c, DestVI. d, CARD. e, NovoSpaRc. f, CellTrek. g, Tangram. Source data are provided as a Source Data file.

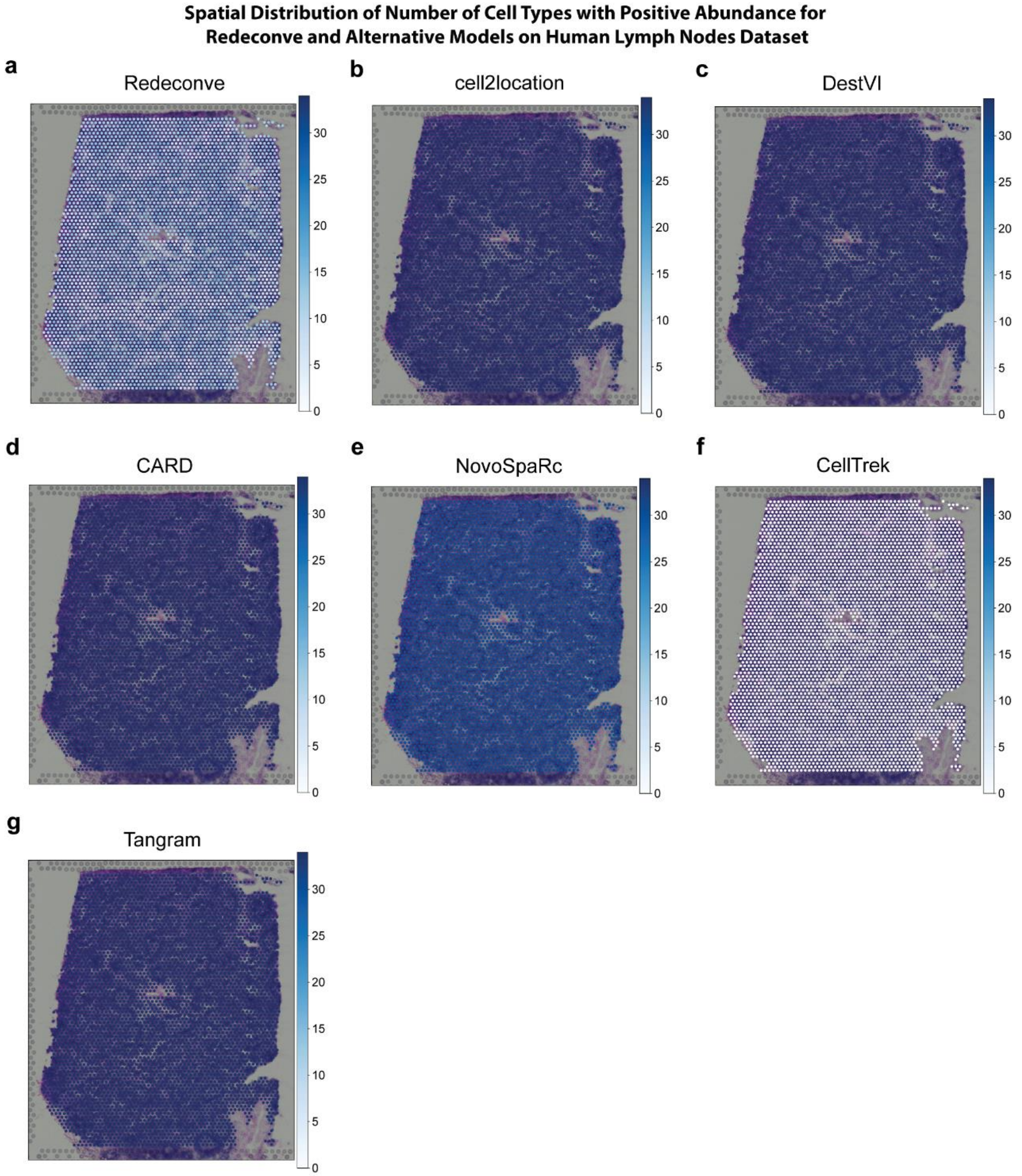

Supplementary Fig 31. Spatial distribution of the number of cell types with positive values for the spots of the human lymph nodes dataset. Color represents the number of cell types with positive values. a, Redeconve. b, cell2location. c, DestVI. d, CARD. e, NovoSpaRc. f, CellTrek. g, Tangram. Source data are provided as a Source Data file.

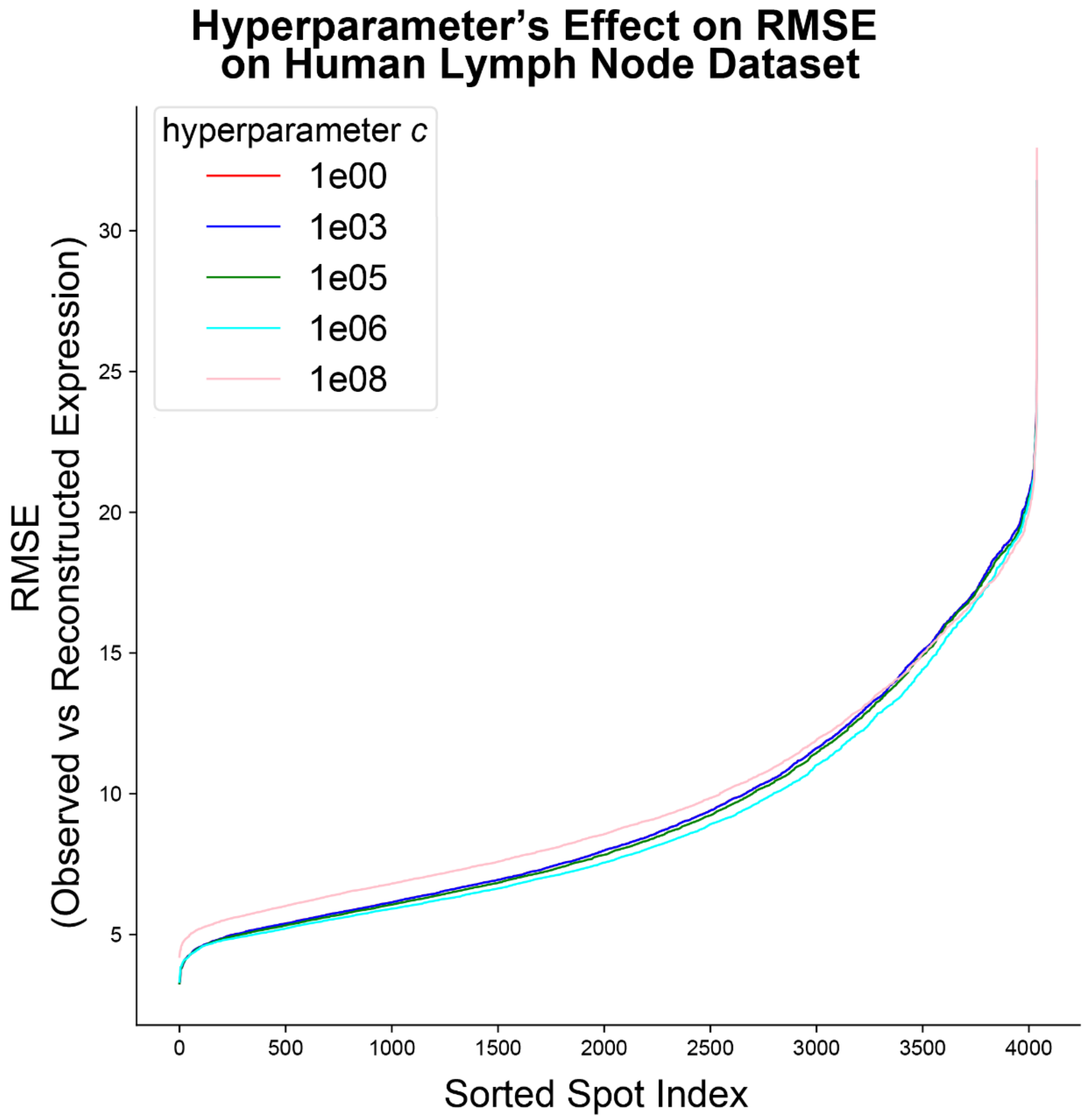

Supplementary Fig 32. Line plot of residuals for different hyperparameters when deconvolution was conducted on the human lymph node dataset by Redeconve. When calculating RMSE_normal, both the predicted and observed expression were first normalized to gene numbers. RMSE, Root-mean-square deviation. Source data are provided as a Source Data file.

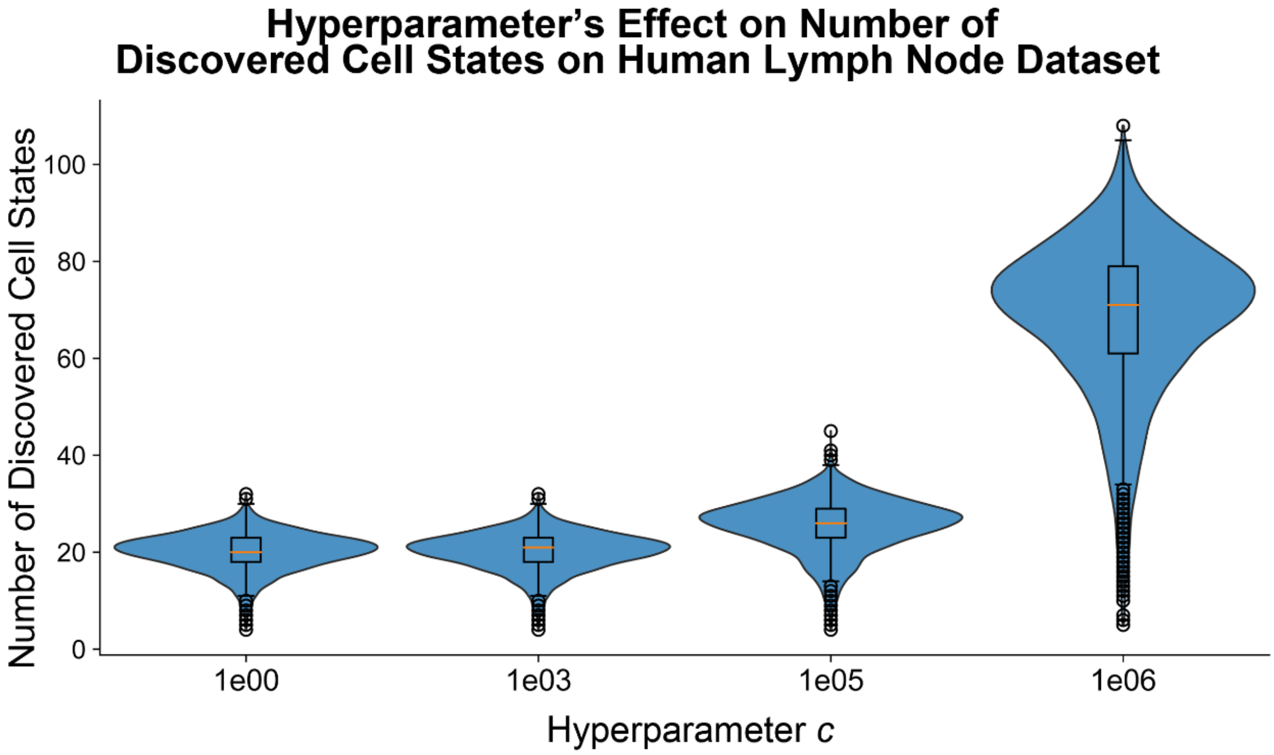

Supplementary Fig 33. Number of discovered cell states in each spot vs. hyperparameter on PDAC dataset. A bigger hyperparameter would lead to more cell states per spot. PDAC, pancreatic ductal adenocarcinoma. Source data are provided as a Source Data file.

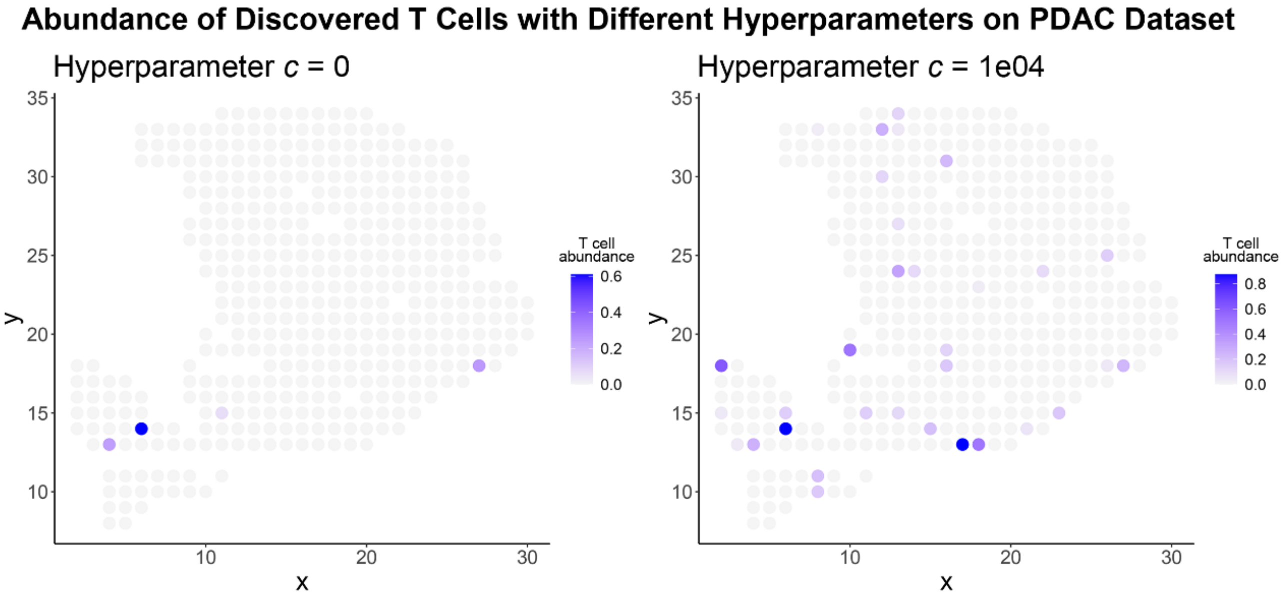

Supplementary Fig 34. Distribution of T cell discovery with different hyperparameters on PDAC dataset. A bigger hyperparameter would lead to more occurrence of certain cells. PDAC, pancreatic ductal adenocarcinoma. Source data are provided as a Source Data file.

Perplexity vs. Abundance or Number of Non-zero Values on Real Datasets

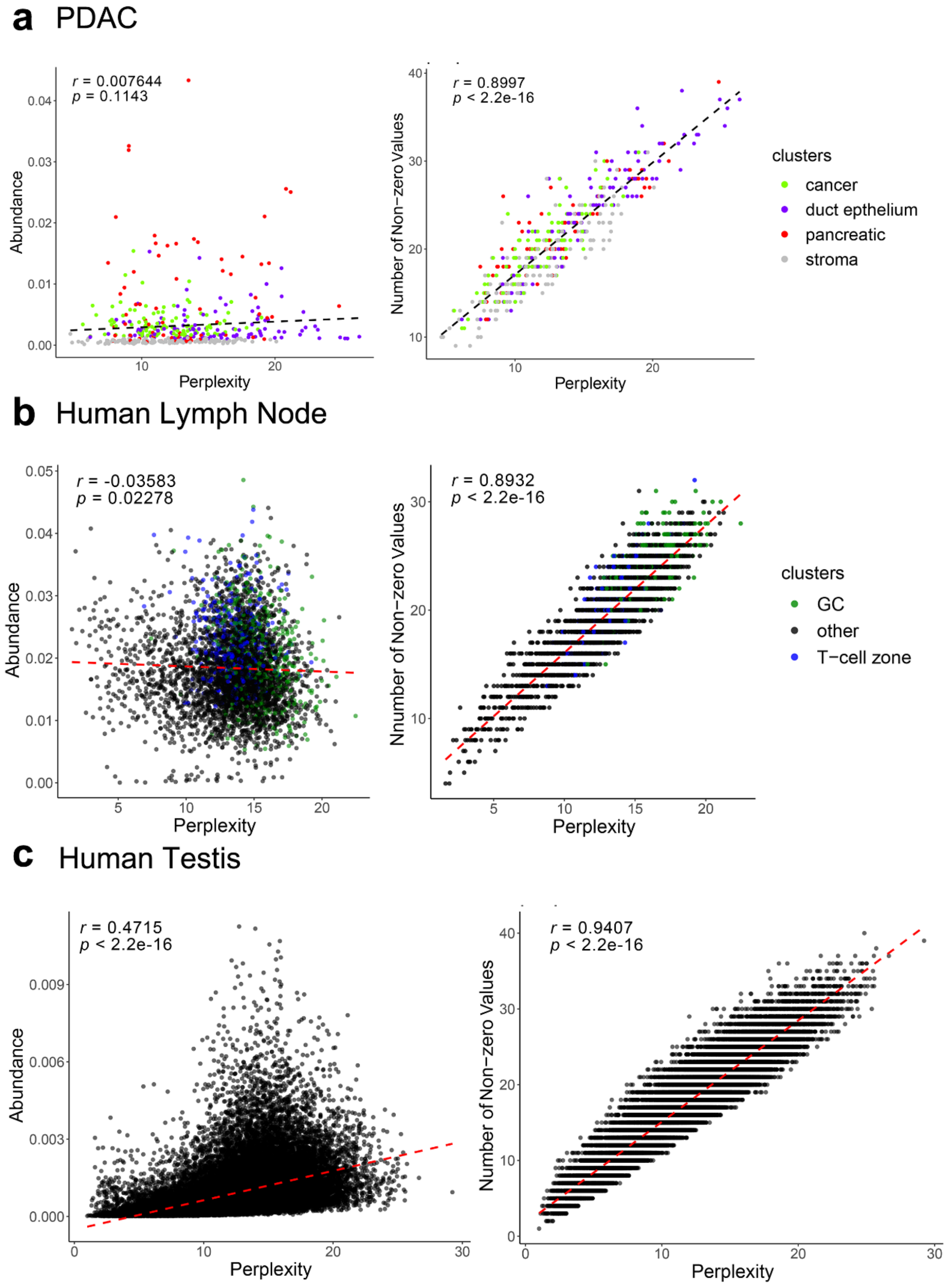

Supplementary Fig 35. Perplexity vs. absolute abundance and number of non-zero values on simulated datasets. Number of non-zero values represents number of cell states in the spot. a, PDAC (pancreatic ductal adenocarcinoma). b, human lymph node. c, human testis. Source data are provided as a Source Data file.

Supplementary Tables

Supplementary Table S1. Perplexity vs. absolute abundance and number of clusters on simulated datasets.

Perplexity vs number of clusters and absolute abundance in simulated PDAC dataset			
	cluster	perplexity	abundance
1	13	11.13	1926
2	31	30.13	1926
3	83	82.14	1926
4	187	180.06	1926
5	318	311.36	1926
0	1926	1926	1926

Perplexity vs number of clusters and absolute abundance in simulated human lymph node dataset			
	cluster	perplexity	abundance
1	10	9.24	1002
2	21	20.12	1002
3	39	38.12	1002
4	85	80.62	1002
5	156	149.05	1002
0	1002	1002	1002

Perplexity vs number of clusters and absolute abundance in simulated human testis dataset			
	cluster	perplexity	abundance
1	13	11.97	999
2	28	26.68	999
3	52	50.17	999
4	113	102.72	999
5	160	145.8	999
0	999	999	999

Supplementary Table S2. Marker genes of cell types for PDAC dataset

**Cell Types**	**Marker Genes**
Acinar	*SYCN, CELA2A, CELA2B, CPA2, CELA3A, CEL, PLA2G1B, CTRB1, CELA3B, CLPS, CTRB2, CPA1, AMY1C, PRSS2, PRSS1, PNLIP, CPB1*
Cancer clone A	*TM4SF1, PITX1, CLDN1, S100A14*
Cancer clone B	*S100A4, SLC20A1, KRT17*
Ductal - APOL1 high/hypoxic	*APOL1, ERO1A, CA9, KRT19*
Ductal - CRISP3 high/centroacinar like	*CRISP3, CFTR, KRT19*
Ductal - MHC Class II	*CD74, HLA-DPA1, HLA-DQA2, HLA-DRA, HLA-DRB1, HLA-DRB5, C1S, C4A, C4B, CFB, CFH, KRT19*
Ductal - terminal ductal like	*TFF1, TFF2, TFF3, KRT19*
Endocrine cells	*CFC1B, SCG3, CFC1, PCSK1, PPY, CHGB, GHRL*
Endothelial cells	*VWF, ACKR1, CLDN5, PLVAP, AQP1*
Fibroblasts	*THBS2, SFRP2, LUM, APOD, COL1A2, MMP11, SFRP4, COL3A1, COL6A3, CCDC80, COL1A1, DCN*
Macrophages A	*IL1B, CD68, IL1RN, CD163*
Macrophages B	*CD163, MS4A4A, CD68, IL1RN*
Mast cells	*KIT, TPSAB1, CPA3, TPSB2*
Monocytes	*CD14, S100A8, S100A9, VCAN, LYZ*
RBCs	*HBD, HBB, HBA2*
T cells & NK cells	*CD3D, CD3E, CD3G, CD8A, CD8B, CCL5, GNLY, CD4, FOXP3*
Tuft cells	*POU2F3, DCLK1, SOX9, CDHR2, ESPN, TSLP, DRD3, AVIL, RGS13*
mDCs A	*RASSF4, C1QA, C1QB, C1QC*
mDCs B	*SLC2A5, C1QA, C1QB, C1QC*
pDCs	*TCL1A, JCHAIN, LILRA4*

Supplementary Table S3. Assigning cell types to clusters in scFFPE-seq data

Cluster IDs	Assigned cell types
1	CD4+ T cells
2	Stromal
3	CD8+ T cells
4	CD8+ T cells
5	DCIS
6	Invasive tumor
7	Macrophages #1
8	DCIS
9	Invasive tumor
10	Invasive tumor
11	Myoepithelial
12	B cells
13	Invasive tumor
14	Invasive tumor
15	Invasive tumor
16	Macrophages #2
17	Endothelial
18	B cells
19	Invasive tumor
20	Stromal
21	Macrophages #2
22	B cells

Supplementary Table S4. Assigning cell types to clusters in Xenium data

Cluster IDs	Assigned cell types
1	Invasive tumor
2	Stromal
3	DCIS
4	Macrophages #1
5	CD4+ T cells
6	CD8+ T cells
7	Invasive tumor
8	Endothelial
9	Invasive tumor
10	CD4+ T cells
11	Stromal
12	Myoepithelial
13	Myoepithelial
14	B cells
15	B cells
16	Myoepithelial
17	Invasive tumor
18	Invasive tumor
19	Invasive tumor
20	Macrophages #2
