# Supplementary material for: Spatial transcriptomics deconvolution at single-cell resolution using Redeconve
Source: Nat Commun. 2023 Dec 1;14:7930. doi: 10.1038/s41467-023-43600-9 (PMC10692090; doi:10.1038/s41467-023-43600-9)
Supplement: Supplementary file 3 — Reporting Summary [file 41467_2023_43600_MOESM3_ESM.pdf]

nature portfolio

Corresponding author(s): Xianwen Ren

Last updated by author(s): Oct 17, 2023

Reporting Summary

Nature Portfolio wishes to improve the reproducibility of the work that we publish. This form provides structure for consistency and transparency in reporting. For further information on Nature Portfolio policies, see our Editorial Policies and the Editorial Policy Checklist.

Statistics

For all statistical analyses, confirm that the following items are present in the figure legend, table legend, main text, or Methods section.

n/a	Confirmed	
☐	☒	The exact sample size (*n*) for each experimental group/condition, given as a discrete number and unit of measurement
☐	☒	A statement on whether measurements were taken from distinct samples or whether the same sample was measured repeatedly
☐	☒	The statistical test(s) used AND whether they are one- or two-sided<br>*Only common tests should be described solely by name; describe more complex techniques in the Methods section.*
☐	☒	A description of all covariates tested
☐	☒	A description of any assumptions or corrections, such as tests of normality and adjustment for multiple comparisons
☐	☒	A full description of the statistical parameters including central tendency (e.g. means) or other basic estimates (e.g. regression coefficient) AND variation (e.g. standard deviation) or associated estimates of uncertainty (e.g. confidence intervals)
☐	☒	For null hypothesis testing, the test statistic (e.g. *F*, *t*, *r*) with confidence intervals, effect sizes, degrees of freedom and *P* value noted<br>*Give P values as exact values whenever suitable.*
☐	☒	For Bayesian analysis, information on the choice of priors and Markov chain Monte Carlo settings
☐	☒	For hierarchical and complex designs, identification of the appropriate level for tests and full reporting of outcomes
☐	☒	Estimates of effect sizes (e.g. Cohen's *d*, Pearson's *r*), indicating how they were calculated

Our web collection on statistics for biologists contains articles on many of the points above.

Software and code

Policy information about availability of computer code

Data collection	No software used in data collection.

Data analysis	Packages/softwares associated with deconvolution:<br>Redeconve (https://codeocean.com/capsule/5481250/tree, https://github.com/ZxZhou4150/Redeconve),<br>cell2location (https://github.com/BayraktarLab/cell2location/)<br>Tangram (https://github.com/broadinstitute/Tangram)<br>NovoSpaRc (https://github.com/rajewsky-lab/novosparc)<br>CellTrek (https://github.com/navinlabcode/CellTrek)<br>DestVI (https://github.com/romain-lopez/DestVI-reproducibility)<br>CARD (https://github.com/YingMa0107/CARD)<br><br>Other packages/softwares associated with analysis (R):<br>R 4.2.1<br>edgeR 3.38.2<br><br>Other packages/softwares associated with analysis (python):<br>cellpose 2.2.2<br>squidpy 1.3.0

For manuscripts utilizing custom algorithms or software that are central to the research but not yet described in published literature, software must be made available to editors and reviewers. We strongly encourage code deposition in a community repository (e.g. GitHub). See the Nature Portfolio guidelines for submitting code & software for further information.

Data

Policy information about availability of data

All manuscripts must include a data availability statement. This statement should provide the following information, where applicable:

- Accession codes, unique identifiers, or web links for publicly available datasets
- A description of any restrictions on data availability
- For clinical datasets or third party data, please ensure that the statement adheres to our policy

All the datasets associated with this study is publicly available, including a human pancreatic ductal adenocarcinomas dataset, a human lymph node dataset, a mouse cerebellum dataset, a human breast cancer Visium dataset, a human testis dataset, a mouse brain dataset and a human breast cancer Xenium dataset.

PDAC: The PDAC data used in this study are available in the Gene Expression Omnibus database under accession code GSE111672 [https://www.ncbi.nlm.nih.gov/geo/query/acc.cgi?acc=GSE111672].
Human lymph nodes: The processed human lymph nodes Visium data are available at 10x Genomics website [https://www.10xgenomics.com/resources/datasets/human-lymph-node-1-standard-1-1-0]. The processed human lymph nodes scRNA-seq data are available from Kleshchevnikov et al. [https://cell2location.cog.sanger.ac.uk/browser.html].
Mouse cerebellum: The mouse cerebellum data used in this study are available in the Single Cell Portal database under accession code SCP948 [https://singlecell.broadinstitute.org/single_cell/study/SCP948/robust-decomposition-of-cell-type-mixtures-in-spatial-transcriptomics#study-download].
Human breast cancer Visium: The processed human breast cancer Visium data are available at zenodo [https://zenodo.org/record/4739739#.Ys0v6jdBy3D]. The processed human breast cancer scRNA-seq data used in this study are available in the Gene Expression Omnibus database under accession code GSE176078 [https://www.ncbi.nlm.nih.gov/geo/query/acc.cgi?acc=GSE176078].
Human testis: The processed human testis Slide-seq data are available at dropbox [https://www.dropbox.com/s/q5djhy006dq1yhw/Human.7z?dl=0]. The processed human testis scRNA-seq data used in this study are available in the Gene Expression Omnibus database under accession code GSE112013 [https://www.ncbi.nlm.nih.gov/geo/query/acc.cgi?acc=GSE112013].
Mouse brain Visium: The processed mouse brain Visium data used in this study are available in the ArrayExpress database under accession code E-MTAB-11114 [https://www.ebi.ac.uk/biostudies/arrayexpress/studies/E-MTAB-11114?accession=E-MTAB-11114]. The processed mouse brain snRNA-seq data used in this study are available in the ArrayExpress database under accession code E-MTAB-11115 [https://www.ebi.ac.uk/biostudies/arrayexpress/studies/E-MTAB-11115?query=E-MTAB-11115].
Xenium: The processed Visium, 3' scRNA-seq, 5' scRNA-seq and scFFPE-seq for human breast cancer Xenium dataset are available at 10x Genomics website [https://www.10xgenomics.com/products/xenium-in-situ/preview-dataset-human-breast].
Source data are provided with this paper.

Human research participants

Policy information about studies involving human research participants and Sex and Gender in Research.

Reporting on sex and gender	This study has no association with human research participants.
Population characteristics	This study has no association with human research participants.
Recruitment	This study has no association with human research participants.
Ethics oversight	This study has no association with human research participants.

Note that full information on the approval of the study protocol must also be provided in the manuscript.

Field-specific reporting

Please select the one below that is the best fit for your research. If you are not sure, read the appropriate sections before making your selection.

[x] Life sciences   [ ] Behavioural & social sciences   [ ] Ecological, evolutionary & environmental sciences

For a reference copy of the document with all sections, see nature.com/documents/nr-reporting-summary-flat.pdf

Life sciences study design

All studies must disclose on these points even when the disclosure is negative.

Sample size	Redeconve is evaluated across 7 spatial transcriptomics datasets, 6 are sequencing-based and 1 is imaging- based. No prior sample-size calculation was performed. We chose those samples because they have high-quality paired sc/snRNA-seq datasets as reference. We believe these samples are sufficient because they covered all major spatial transcriptomics platforms.
Data exclusions	For spatial transcriptomics data, all the spots were used. For sc/sn RNA-seq data as reference, we down sampled to around 1000 cells. Stratified sampling was performed when cell types are available, otherwise simple random sampling was performed. The exact number of chosen cells for each dataset are as follows: human breast cancer: 1001, human lymph nodes: 1000, human testis: 999, Mouse Brain: 1003, Mouse cerebellum: 1003, human breast cancer Xenium (scFFPE): 1001, human breast cancer Xenium (3'): 998, human breast cancer Xenium (5'): 1002. The seed was set to 2233. In the PDAC dataset, all the 1926 cells in reference were used.
Replication	We repeated each calculation three times to verify the reproducibility. Redeconve got exactly the same results namely, the attempt at replication is successful. Cell2loction and DestVI failed the attempt at replication because they are non-deterministic methods.
Randomization	Randomization is involved only in down sampling sc/snRNA-seq reference as described in "data exclusions" section. For other parts of this study, randomization is not included because each sample was analyzed separately.
Blinding	No blinding is included in this study because we did not include comparison of case/control groups in this study.

Reporting for specific materials, systems and methods

We require information from authors about some types of materials, experimental systems and methods used in many studies. Here, indicate whether each material, system or method listed is relevant to your study. If you are not sure if a list item applies to your research, read the appropriate section before selecting a response.

Materials & experimental systems

n/a	Involved in the study
[x]	[ ] Antibodies
[x]	[ ] Eukaryotic cell lines
[x]	[ ] Palaeontology and archaeology
[x]	[ ] Animals and other organisms
[x]	[ ] Clinical data
[x]	[ ] Dual use research of concern

Methods

n/a	Involved in the study
[x]	[ ] ChIP-seq
[x]	[ ] Flow cytometry
[x]	[ ] MRI-based neuroimaging
